# Understanding the Scope, Intent and Extent of Published Conceptual Frameworks of Frameworks for Patient and Public Involvement in Health and Social Care Research: A Rapid Scoping Review

**DOI:** 10.1111/hex.70425

**Published:** 2025-09-04

**Authors:** Eugenie Evelynne Johnson, Sean Gill, Madeleine Still, Daisy Trenchard, Debbie Smith, Rebecca Harmston, Jane McDermott, Fiona Pearson

**Affiliations:** ^1^ NIHR Innovation Observatory Newcastle University Newcastle upon Tyne UK; ^2^ Population Health Sciences Institute Newcastle University Newcastle upon Tyne UK; ^3^ Public Research Team Member UK

**Keywords:** patient engagement, patient involvement, public involvement and engagement, scoping review

## Abstract

**Introduction:**

The United Kingdom National Institute for Health and Care Research (NIHR) allocates funding and provides infrastructure, training and capacity building for research. NIHR expects that patient and public involvement (PPI) is embedded within research it supports. There is a need to understand more about what guidance is offered to researchers across PPI frameworks. This rapid scoping review aimed to identify and clarify PPI frameworks for health and care research.

**Objective:**

To identify and explore the scope and key features of frameworks for PPI in health and social care research.

**Methods:**

We undertook a rapid scoping review, conducing searches on MEDLINE, CINAHL and PsycInfo for relevant records indexed from 2013 to August 2024. After piloting to refine eligibility and ensure consistent decision‐making, a single reviewer screened titles and abstracts and then full‐texts, with another checking a proportion for accuracy. A data charting form was piloted. Two reviewers charted all eligible frameworks, and a third checked accuracy. We synthesised data using graphs and tables and provided a narrative of results.

**Results:**

We included 53 frameworks from 55 reports. Most suggested they were applicable across types of health or social care research (*N* = 30), influencing different stages of the research process (*N* = 39). Most were developed in the UK (*N* = 28). Most frameworks did not specify how to find patients or members of the public (*N* = 36), whether PPI should be one‐time or continuous (*N* = 34), or how direct any interaction between patients and the public and researchers should be (*N* = 33). Eighteen frameworks suggested that patients and the public could have different levels of control over research. Most frameworks (*N* = 49) suggested ways to meet one or more of the UK Standards for Public Involvement. Few suggested ways in which equity or diversity could be considered in PPI, according to PROGRESS‐Plus domains.

**Conclusions:**

Future frameworks should provide clear, practical guidance to researchers on how to involve people in different types of health and social care research, including how to approach different groups and consider equity and inclusivity within PPI.

**Abbreviations**
ACTIVE: Authors and Consumers Together Impacting on eVidencE
JBI: Joanna Briggs Institute
NICE: National Institute for Health and Care Excellence
NIHR: National Institute for Health and Care Research
PPI: patient and public involvement
PRISMA: Preferred Reporting Items for Systematic reviews and Meta‐Analysis
PRISMA‐ScR: Preferred Reporting Items for Systematic reviews and Meta‐Analysis for Scoping Reviews
UK: United Kingdom

## 1 Introduction

The National Institute for Health and Care Research (NIHR) in the United Kingdom promotes patient and public involvement (PPI) within health and care research in the UK. The NIHR describes involvement as research carried out ‘with’ or ‘by’ patients or members of the public, rather than ‘about’ them [1]. The benefits of PPI within health research for patients, members of the public and researchers have been well documented, including: enhancing relevance of research; improving overall quality; increasing the reach of findings; improving knowledge of research amongst the public; and growing patient empowerment [2].

Currently, there are many published frameworks to guide approaches to PPI in health and care research; in 2019, a review included 65 such frameworks [3]. However, researchers still express the need for practical support, training and resources to undertake PPI in their work and identified frameworks were found to have limited transferability. Indeed, a 2019 qualitative study exploring health researchers’ attitudes to PPI noted that responding researchers often felt ‘ill‐equipped’ in terms of resources and training for PPI [4]. Furthermore, a systematic review and thematic analysis in the specific context of evidence synthesis also highlighted the need for sufficient training and support, including appropriate materials and resources to guide PPI [2], while a cross‐sectional survey surrounding PPI in statistical methodology research also highlighted that respondents would find guidance on how to conduct PPI in this context helpful [5].

As such, there appears to be a mismatch between the growing number of resources to aid the undertaking of PPI being published and the perspectives of those looking to embed PPI into health and care research. Some prior published works have already explored the similarities and differences in PPI frameworks. In 2019, a review was undertaken to identify and synthesise published frameworks to support PPI in health research, but used a hermeneutic approach of accessing and interpreting the literature and developing an argument, placing a major focus on creating an overarching taxonomy for the frameworks identified and highlighting whether they were being used in practice [3]. Furthermore, a scoping review published in 2022 examined the different elements that comprise models and frameworks for PPI, but only in the specific, narrow context of health services research [6]. We therefore need to understand more about what implementable guidance is being offered to researchers across available PPI frameworks within the broader context of health and care research.

This study aims to identify and explore the scope, common concepts and features and potential inclusivity of conceptual frameworks for conducting and reporting PPI across the broad area of health and social care research.

## 2 Methods

Conduct was in accordance with the JBI methodology for scoping reviews [7]. The protocol was published on the Open Science Framework on 18 July 2024 with deviations from this documented in Supporting Information S1 [8]. Definitions of key terms used within the methods and results of this paper are presented in Table 1.

**Table 1:** Definitions of key terms used.

Topic	Term	Definition
Frameworks	ACTIVE framework	A framework used to help describe how people how patients and members of the public can be involved in systematic reviews. Systematic reviews are a type of evidence synthesis [9].
	Framework	For the purposes of this review, a ‘framework’ tells researchers how they can involve patients and the public in health and care research. This did not include individual case studies about involving people in research.
	PROGRESS‐Plus	PROGRESS‐Plus is a way of identifying factors reported in studies that are often linked to health and wellbeing. This includes where people live, their occupation, ethnicity, religion, sex/gender and other personal characteristics, such as age and disability. It can be used to find out whether research has thought about factors that might lead to health inequalities [10, 11].
Approaches to patient and public involvement	Fixed approach to recruitment	A fixed approach to finding people means that only specific people are asked to be involved in the research. This includes asking people researchers already know or recruiting from an existing group. It could also mean asking people to be involved if they have a specific health condition or are a member of a group of patients that the researcher is interested in [9].
	Open approach to recruitment	An open approach to recruitment means providing opportunities that allow anyone to be involved in research. This approach can either be ‘fixed’ or ‘flexible’. ‘Fixed’ means the people involved are chosen once and stay the same. ‘Flexible’ means the people involved can change over the course of a research project [9].
	Patient and public involvement	The National Institute for Health and Care Research (NIHR) in the UK defines patient and public involvement as research that is carried out ‘with’ or ‘by’ members of the public rather than ‘to’, ‘about’ or ‘for’ them [1].
	Patient and public engagement	The National Institute for Health and Care Research (NIHR) in the UK defines patient and public engagement in research as the practice of sharing and providing information and knowledge from research to a wider audience [1].
	UK Standards for Public Involvement	The UK Standards for Public Involvement are a set of six principles designed to improve the quality and consistency of public involvement in research. The standards are: communication; inclusive opportunities; training and support; governance; working together; and impact [12].
Evidence synthesis	Evidence synthesis	A type of research that gathers information from different sources to try and answer a specific question. The sources are usually published studies or reports. Evidence syntheses can answer a range of questions, including how well a particular treatment works, what people's experiences are of a condition, or how accurate a diagnostic test is.
	Population, Concept, Context (PCC)	The term ‘Population, Concept, Content’, or ‘PCC’, is a way of deciding what sources of information should be included in a scoping review. It lets people know who is eligible to be in the review and what ideas it will focus on. This can include treatments for a health condition. It might also be about the results of research studies or how the studies were done. It also lets people know if the literature should come from a particular area, country or health and care setting [13].
	Scoping review	A scoping review is a type of evidence synthesis used to look at all the evidence on a specific type of research question. It used a clear and planned approach to find, select and analyse studies. They are often about understanding what we know or do not know about a specific topic [13].

### 2.1 Patient and Public Involvement

Two members of the public (D.S. and R.H.) helped govern the conduct of the scoping review in accordance with the UK Standards for Public Involvement [12, 14]. Both members of the public had previously worked with the lead reviewer [15], as well as being experienced in public involvement in research through other activities. This meant they required less support and training to be involved, though key concepts surrounding the methods used were explained in plain language where appropriate. They were given a brief introduction to the wider project that included this scoping review and helped govern the project from a public perspective by attending research team meetings and participating in discussions surrounding the conduct and findings of the review. Their time working on the project was reimbursed at NIHR‐recommended rates [16]. Further details on how they contributed to this project are documented within this report and in Supporting Information S2.

### 2.2 Eligibility Criteria

The full eligibility criteria for the review are described in Supporting Information S3. In brief, we used the Population, Concept, Context framework to shape the eligibility criteria. We considered any framework aiming to aid the conduct of PPI in health and social care research, regardless of the research method, the population of interest (including demographic and cultural factors) or contextual factors (such as clinical setting or geography) [13]. Studies published in any language and published since 2013 were included. To be eligible, frameworks had to provide guidance on how to approach and operationalise involving patients and the public in health and social care research. We did not include frameworks that provide guidance solely on how to engage with them. To make this distinction between involvement and engagement, we used the definitions provided by the NIHR [1]. However, frameworks could be eligible for inclusion in the review if they included guidance on both involvement and engagement.

### 2.3 Search Strategy

A three‐step search strategy was utilised in this review. First, an initial limited search of MEDLINE (Ovid) and CINAHL (EBSCO) was undertaken to identify articles on the topic. The text words contained in the titles and abstracts of relevant articles, and the index terms used to describe the articles, were used to develop a full search strategy for CINAHL (EBSCO), MEDLINE and PsycInfo (via Ovid; see Supporting Information S4).

We also searched: NIHR Learning for Involvement; the King's Fund; the Royal Colleges of Physicians, Nursing and Midwifery; and the National Co‐ordinating Centre for Public Engagement. The search strategy, including all identified keywords and index terms, was adapted for each included bibliographic information source (see Supporting Information S4). In brief, three independent reviewers (E.E.J., S.G. and D.T.) searched the websites detailed in ‘Search strategies’ to identify potentially relevant articles and resources using the terms ‘framework’, ‘guideline’, ‘guidance’, ‘evaluation’ and ‘policy’. We performed backwards and forwards citation chaining on relevant systematic reviews.

### 2.4 Study Selection

We collated, deduplicated and uploaded all identified citations into Rayyan [17]. Three reviewers (E.E.J., S.G. and D.T.) conducted a pilot screen on 10% of records. Following this, three independent reviewers (E.E.J., S.G. and D.T.) assessed remaining titles and abstracts for eligibility. Potentially relevant sources were retrieved in full and were assessed in detail against the eligibility criteria by one reviewer in Rayyan (E.E.J.), with 50% checked by a second reviewer (S.G.) [17]. Any disagreements were resolved by discussion or, if necessary, through arbitration to a third reviewer (D.T.). At both stages, if disagreement between the reviewers had been deemed too high (20% or more), we would have discussed the eligibility criteria further and refined them until adequate agreement was reached.

Potentially relevant records were copied into Excel and further assessed, with reasons for exclusion provided where applicable. Two independent reviewers (E.E.J. and M.S.) independently assessed the full text of 50% of the records identified by citation chaining each, checking 20% of the other's decisions for accuracy.

Had we found potentially relevant records published in a language other than English, we would have attempted to obtain a translation to determine eligibility. We have presented the results of the search and the full study inclusion process in the PRISMA flow diagram [18].

### 2.5 Data Charting

We created a data charting form within Microsoft Excel. Three independent reviewers (E.E.J., S.G. and M.S.) piloted the form on 10% of included records. Following piloting, we modified and revised the draft data charting tool. The final data charting form contained items on: bibliographic details; population; context; how PPI is proposed to be recruited, approached and involved (adapted from the ACTIVE framework) [9]; which of the UK Standards for Public Involvement are considered by the framework [12, 14]; and any PROGRESS‐Plus criteria considered by the framework [10, 11]. Full details are provided in Supporting Information S5.

Following piloting, two independent reviewers (E.E.J. and S.G.) charted 50% of the remaining records each. One of two independent reviewers (E.E.J. or S.G. as appropriate) checked the charted data for accuracy. Any disagreements were resolved through discussion. If required, we would have contacted study authors to request additional data.

We grouped multiple included records for a single PPI framework into a single unit of analysis, charting data for a primary record with the most information and using the remaining allied records to supplement the charting with additional information [19].

### 2.6 Data Analysis and Presentation

Following data charting, we analysed data in Microsoft Excel and presented results in tabular or graphical format with an accompanying narrative synthesis. In doing so, we reflected on guidance from the JBI regarding presentation of scoping review results [19]. Had data allowed, we would have performed subgroup analyses by the type of health research the PPI was targeted at, stratifying by primary and secondary research. We reported the review in accordance with the PRISMA extension for scoping reviews (PRISMA‐ScR; see Supporting Information S6) [20].

## 3 Results

### 3.1 Results of the Search

**Figure 1:**
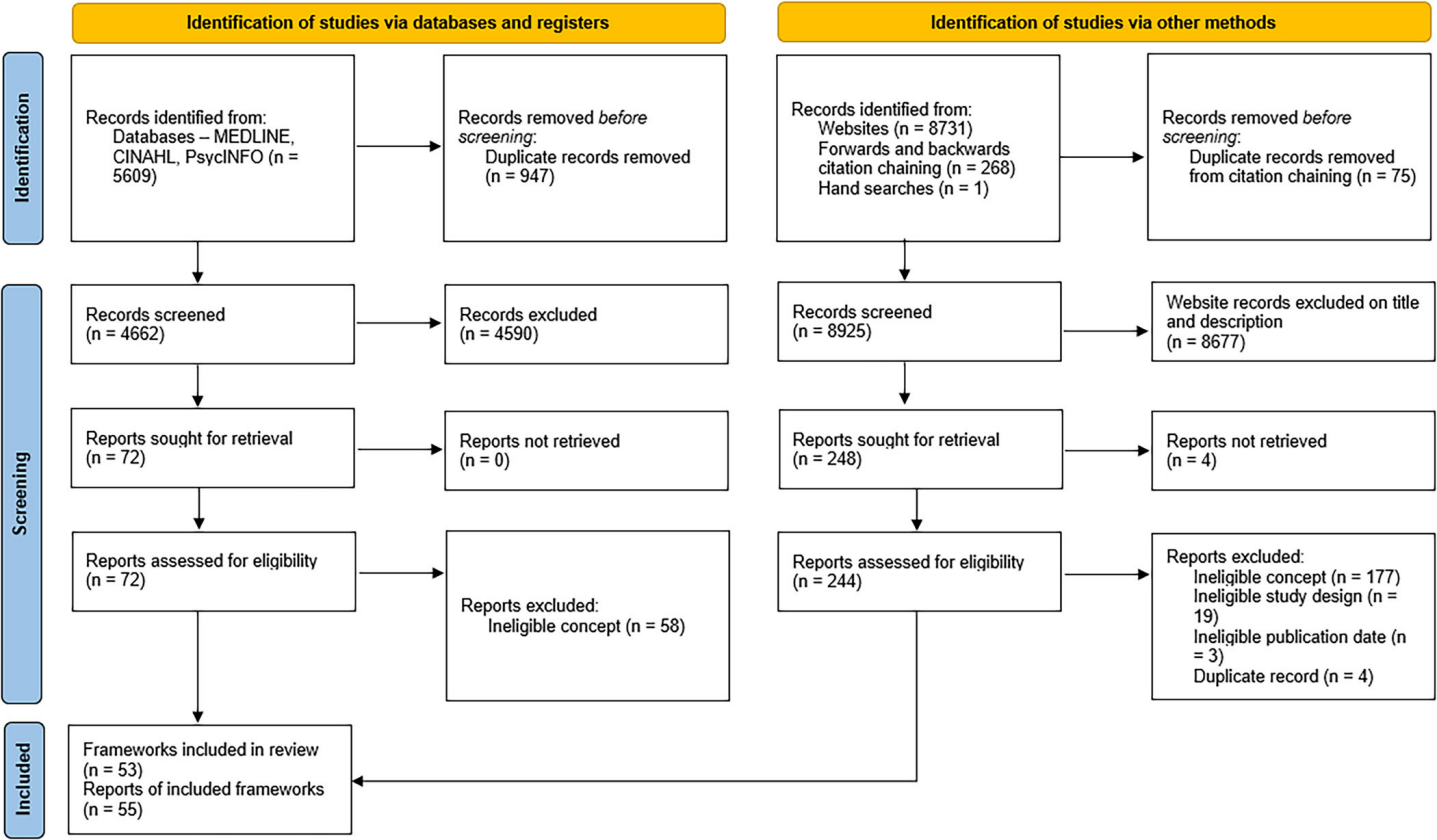
PRISMA flow diagram.

A PRISMA flow diagram can be found in Figure 1 [18]. In total, we included 55 reports of 53 frameworks in this scoping review. A full list of records excluded at full text is provided in Supporting Information S7.

### 3.2 Characteristics of Included Frameworks

Characteristics of included frameworks are presented in Table 2. In brief, 30 of the 53 frameworks, suggested that they could be applied to any form of health and social care research [1, 12, 24, 25, 28, 30, 31, 33, 35, 36, 37, 38, 39, 41, 42, 45, 46, 48, 50, 51, 52, 53, 55, 56, 57, 59, 61, 63, 64, 68, 69, 70], with many developed in the UK (*N* = 28) [1, 9, 12, 22, 30, 32, 35, 36, 37, 38, 41, 42, 47, 48, 51, 53, 54, 55, 56, 57, 59, 60, 61, 62, 63, 66, 67, 69]. Thirty of the 54 frameworks were designed to be used with any patient or member of the public [1, 9, 12, 21, 22, 24, 26, 31, 32, 34, 37, 38, 39, 41, 43, 44, 45, 46, 47, 49, 50, 51, 54, 55, 57, 59, 61, 63, 64, 65, 69, 71], while the remaining 23 were targeted at involving a specific population (e.g., a specific age group or people with a specific condition). Thirty‐nine of the frameworks suggested that they could be used to inform multiple stages of the research process [1, 9, 12, 21, 22, 24, 25, 27, 28, 29, 30, 31, 32, 33, 34, 35, 36, 39, 40, 43, 44, 45, 46, 48, 49, 52, 53, 54, 56, 57, 58, 60, 61, 65, 66, 67, 69, 70, 71, 72].

**Table 2:** Characteristics of included frameworks.

Study ID	Source type	Type of health or social care research	Country/countries of development	Purpose of framework	Part of the research process framework aimed at	Target population
Abelson 2016 [21]	Journal article	HTA	Canada	To involve patients and the public in the government's HTA process	Multiple stages	General population
Bagley 2016 [22]	Journal article	Clinical trials	UK	To support clinical trial teams in a clinical trials unit in undertaking PPI	Multiple stages	General population
Camello Castillo 2015 [23]	Conference abstract	Patient‐centred outcomes research	USA	To assist in including the perspectives of Latin American caregivers in patient‐centred outcome research	Other	Specific population: Hispanic/Latin American caregivers
Canadian Institutes of Health Research (CIHR) 2018 [24]	Webpage	Any	Canada	To establish key concepts, principles and areas for patient engagement to be adopted by a strategy for patient‐oriented research partners	Multiple stages	General population
Charalambous 2023 [25]	Journal article	Any	Multiple countries	To present a framework for guiding patient involvement in aphasia research	Multiple stages	Specific population: people with aphasia
Coulston 2024 [26]	Journal article	Online intervention or service design	Australia	To present a framework for authentically adapting health research co‐design into an online environment	Other	General population
De Wit 2015 [27]	Journal article	Any (specific to rheumatology research)	Netherlands	To present five practical components that can enable equal collaboration between patients and professionals in rheumatology research	Multiple stages	Specific population: rheumatology
Desborough 2022 [28]	Journal article	Any	Australia	To involve young people with Type 1 diabetes in research using a co‐production approach	Multiple stages	Specific population: young people with Type 1 diabetes
Deverka 2018 [29]	Journal article	Clinical trials	USA	To involve people with cancer in one of the largest cancer clinical trial network groups in the USA	Multiple stages	Specific population: people with cancer
Di Lorito 2018 [30]	Journal article	Any	UK	To propose good practice in co‐researching with carers of people with dementia	Multiple stages	Specific population: carers of people with dementia
Etchegary 2022 [31]	Journal article	Any	Canada	To co‐design and operationalise a patient engagement plan for any health research project using a seven‐step approach^a^	Multiple stages	General population
Evans 2013 [32]	Journal article	Clinical trials	UK	To support researchers in involving service users in trials and rigorous studies with a standard operating procedure	Multiple stages	General population
eYPAGnet [33]	Webpage	Any	Multiple countries (across Europe)	To provide information on how to set up a YPAG to support and be involved in research	Multiple stages	Specific population: young people
Fagan 2016 [34]	Journal article	Comparative effectiveness research	USA	To present a pragmatic framework of collaborative engagement and partnership between researchers and people from existing patient and family advisory councils at an academic medical centre	Multiple stages	General population
Faluyi 2024 [35]	Journal article	Any	UK	To address racial equality in public involvement	Multiple stages	Specific population: Black African‐, Asian‐ and Caribbean‐heritage communities
Farooqi 2022 [36]	Journal article	Any	UK	To provide a practical toolkit to help researchers maximise the recruitment of BAME groups in research involvement	Multiple stages	Specific population: BAME communities
Gibson 2017 [37, 38]	1. YouTube video (webinar) 2. Journal article	Any	UK	To present a theoretical framework as a tool for mapping and evaluating the experience of PPI in health services research	Dissemination/impact	General population
Hamilton 2018 [39]	Journal article	Any	Canada	To provide an empirically‐based conceptual framework for patient engagement in research (PEIR) founded on a patient perspective	Multiple stages	General population
Haroutounian 2024 [40]	Journal article	Clinical trials	USA	To provide a framework for meaningful and authentic engagement of patient partners in clinical pain research	Multiple stages	Specific population: people with targeted pain condition(s)
Hoddinott 2018 [41]	Pre‐print journal article	Any (specifically when applying for funding)	UK	To discuss how researchers can involve patients when they are applying for research funding	Other	General population
Jameson 2023 [42]	Journal article	Any	UK	To promote good practice for more inclusive community involvement of racially marginalised community groups in health research	Not reported	Specific population: racially marginalised community groups
Japan Agency for Medical Research and Development 2022 [43]	Report	Clinical trials	Japan	To serve as a guidebook for researchers conducting PPI and to help guide patients and the public who are involved in health research	Multiple stages	General population
Kwon 2018 [44]	Journal article	Patient‐centred outcomes research	USA	To provide guidance on how to apply the core principles of community‐based participatory research (CBPR) in developing patient‐centred outcomes research	Multiple stages	General population
McLean 2023 [45, 46]	1. Journal article 2. Book chapter	Any	Multiple countries (South Africa and Canada)	To provide a framework to help researchers evaluate co‐production	Multiple stages	General population
McNichol 2014 [47]	Journal article	Qualitative or quantitative	UK	To present a series of questions focused around ensuring dissemination of PPI is planned and takes place throughout a project	Dissemination/impact	General population
Mitchell 2019 [48]	Journal article	Any	UK	To help facilitate ethical PPI	Multiple stages	Specific population: children and young people
Murtagh 2021 [49]	Journal article	Genomic science research and implementation	Multiple countries (UK, Australia, USA, Canada, South Africa and Botswana)	To provide a series of questions to support researchers to consider PPIE in their work	Multiple stages	General population
NCCPE 2020 [50]	Report	Any	UK	To aid in designing and delivering online meetings and events	Not reported	General population
NCCPE 2024 [51]	Webpage	Any	UK	To introduce the four pillars of evaluation and how these can be applied	Other	General population
Nicolaidis 2019 [52]	Journal article	Any	USA	To provide a guideline for including autistic adults as both research team members and as study participants	Multiple stages	Specific population: autistic adults
NIHR 2019 [12]	Webpage	Any	UK	To present six value‐based areas to consider when planning and conducting PPI activities in health and care research, showing what ‘good’ PPI looks like and offering reflective questions for practitioners	Multiple stages	General population
NIHR 2020 [53]	Report	Any (COVID‐specific research)	UK	To help facilitate best practice in the design, funding approval, regulation and delivery of COVID‐19 research	Multiple stages	Specific population: groups who may be under‐served due to the COVID‐19 pandemic
NIHR 2021a [54]	Webpage	Clinical trials	UK	To provide definitions of research oversight groups, describes the public member role and expectations and outlines good practice for recruitment and involvement of public members	Multiple stages	General population
NIHR 2021b [55]	Webpage	Any	UK	To provide a framework for considering who might be involved in research and why	Other	General population
NIHR 2021c [56]	Webpage	Any	UK	To present top tips for involving children and young people in health research	Multiple stages	Specific population: children and young people
NIHR 2021d [1]	Webpage	Any	UK	To present the UK standards for public involvement plus links to additional supporting resources	Multiple stages	General population
NIHR 2024 [57]	Webpage	Any	UK	To describe co‐production within research practices	Multiple stages	General population
Oveisi 2024 [58]	Journal article	Qualitative research	Canada	To provide a framework for meaningful engagement with adolescent and young adult cancer patients	Multiple stages	Specific population: adolescent and young adult cancer patients
Pandya‐Wood 2017 [59]	Journal article	Any research, particularly research applications being developed for national peer‐reviewed funding competitions	UK (specifically England)	To assist researchers in recognising ethical issues when involving the public	Study design	General population
Parkinson's UK [60]	Report	Basic science, lab‐based research	UK	To explain and guide how patient involvement can be used within the research cycle, specifically in basic science and lab‐based research	Multiple stages	Specific population: people affected by Parkinson's disease
Pollard 2015 [61]	Journal article	Any	UK	To present guidance for including PPI in research	Multiple stages	General population
Pollock 2019 [9]	Journal article	Systematic reviews	UK	To provide a structure with which to describe key components of stakeholder involvement within a systematic review	Multiple stages	General population
Pollock 2013 [62]	Journal article	Setting research priorities	UK	To facilitate involvement through targeted engagement and assisted involvement to gather research priorities from people affected by stroke using a new model	Other	Specific population: people with stroke
Popay 2014 [63]	Webpage	Multiple	UK	To use during the time research ideas and funding proposals are being developed to assess the impact of public involvement	Study design	General population
Roche 2020 [64]	Journal article	Multiple	Canada	To include a trauma‐informed, intersectional, approach in patient engagement	Not reported	General population
Silva 2021 [65]	Journal article	HTA	Multiple countries (Brazil, UK, Canada)	To provide a framework for PPI in HTA, specifically within the Brazilian context	Multiple stages	General population
Swarbrick 2016 [66]	Journal article	Any (specifically, research with dementia patients and carers)	UK	To facilitate co‐design and co‐leading a research project with people affected by dementia.	Multiple stages	Specific population: people with dementia
Temple 2021 [67]	Webpage	Any (specifically, research with young people)	UK	To provide tips to facilitate better research involvement with young people	Multiple stages	Specific population: young people aged 16 ‐ 25 years, initially with lived experience of mental ill health
Ward 2020 [68]	Journal article	Multiple	Canada	To develop a CBPR framework for health research with the Innu peoples	Other	Specific population: Innu people
Wellcome Trust [69]	Webpage	Multiple	UK	To prompt reflection when planning research through questions	Multiple stages	General population
Weiler‐Wichtl 2024 [70]	Journal article	Any (but specific to oncology‐based research)	Austria	To develop a framework for involving paediatric oncology patients in research	Multiple stages	Specific population: paediatric oncology patients
Wilson 2018 [71]	Journal article	Patient‐centred outcomes in medical product development	USA	To provide a methodological framework for engaging patients at various stages of developing clinical outcome assessments for medical product development	Multiple stages	General population
Wright Nee Blackwell 2016 [72]	Journal article	Emergency department research	USA	To present crucial aspects of patient engagement that are essential for future research in the emergency department	Multiple stages	Specific population: people recruited from emergency departments

Abbreviations: ACTIVE, Authors and Consumers Together Impacting on eVidencE; BAME, black and minority ethnic; CBPR, community‐based participatory research; CIHR, Canadian Institutes of Health Research; CYP, children and young people; HTA, Health Technology Assessment; NIHR, National Institute for Health and Care Research; PEIR, patient engagement in research; PPI, patient and public involvement; PPIE, patient and public involvement and engagement; UK, United Kingdom; USA, United States of America; YPAG, Young people's advisory group.

^a^ ‘Engagement’ in this context is involvement following NIHR definitions.

## 4 Main Results

Very few of the included frameworks reported on some of the practical details surrounding PPI as described by the ACTIVE framework [9]. Thirty‐six of the 53 frameworks did not propose a specific method of recruiting patients and the public to be involved in research. Of the remaining 17 that did report on recruitment methods, six suggested an open and fixed approach [22, 25, 27, 32, 41, 54], five suggested an open and flexible approach [33, 42, 57, 61, 71], one suggested direct invitations [70], two suggested approaching an existing group [34, 68], while three suggested more than one of these methods of recruitment [9, 64, 72].

In terms of length of involvement, 34 of 53 included frameworks did not report whether PPI should be one‐time or continuous. Of the frameworks that did report this information, 16 suggested continually involving patients and the public in research [22, 25, 28, 29, 30, 31, 32, 34, 40, 42, 45, 46, 54, 57, 60, 68, 70], two suggested one‐time PPI in research [33, 62, 65] and one suggested a mixture of the two methods [9].

Thirty‐three of the 53 included frameworks did not suggest whether there should be direct interaction, multiple discrete events or no direct interaction between researchers and patients or members of the public when undertaking research involvement activities. Of the remaining 19 frameworks, 9 suggested interacting with patients and the public directly [26, 30, 33, 42, 50, 57, 62, 68, 70], while 11 suggested a combination of both direct and indirect interactions [9, 21, 22, 25, 31, 43, 52, 54, 58, 60, 71]. Twelve of the frameworks described employing both face‐to‐face and digital interactions (e.g., via Zoom or Microsoft Teams) to interact with patients or members of the public [9, 21, 22, 31, 33, 43, 52, 54, 58, 60, 71]. Four proposed only face‐to‐face interaction [12, 42, 62, 68], two were specifically aimed at influencing online involvement [26, 50], while two did not specifically report on interaction method [25, 57].

Nineteen of the 53 included frameworks did not report on the level of involvement that patients and the public should have within a research project. However, 18 frameworks suggested that patients and the public could have multiple different levels of involvement in the research process [1, 9, 21, 22, 27, 30, 31, 37, 38, 41, 52, 58, 60, 61, 63, 65, 68, 71, 72]. Of those that only posited one level of involvement, four suggested that patients and the public should be leading research [24, 44, 45, 46, 57], four suggested they should be controlling research [25, 26, 34, 54], three reported involvement at the influencing level [29, 32, 33], four as contributors [53, 59, 62, 70] and one where patients and the public were receivers of information only [50].

**Figure 2:**
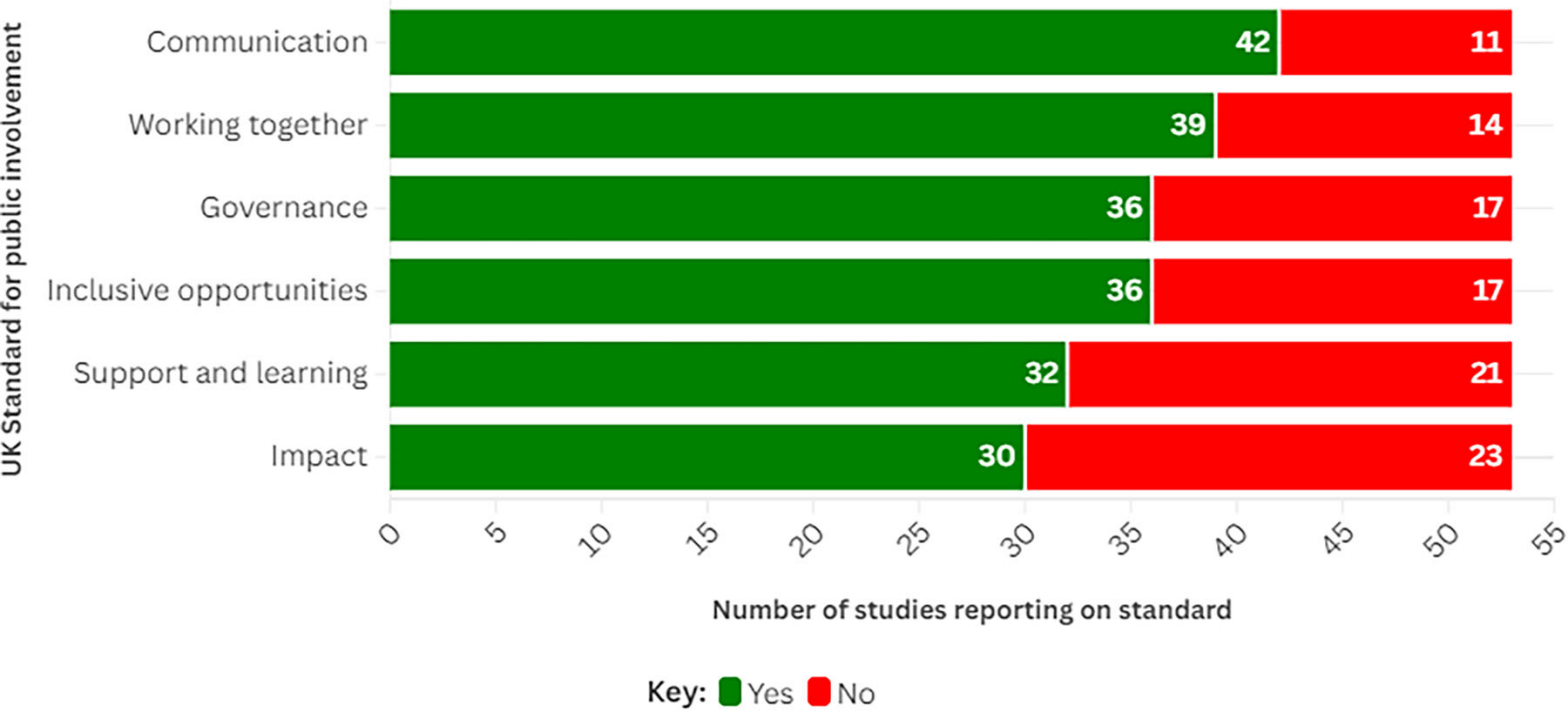
UK Standards for public involvement reported by identified frameworks.

An overview of how many identified frameworks suggested strategies that would help researchers to meet each of the components of UK Standards for public involvement is shown in Figure 2. All but four of the included frameworks reported on an aspect of at least one of the UK Standards for Public Involvement [49, 64, 66, 69].

**Figure 3:**
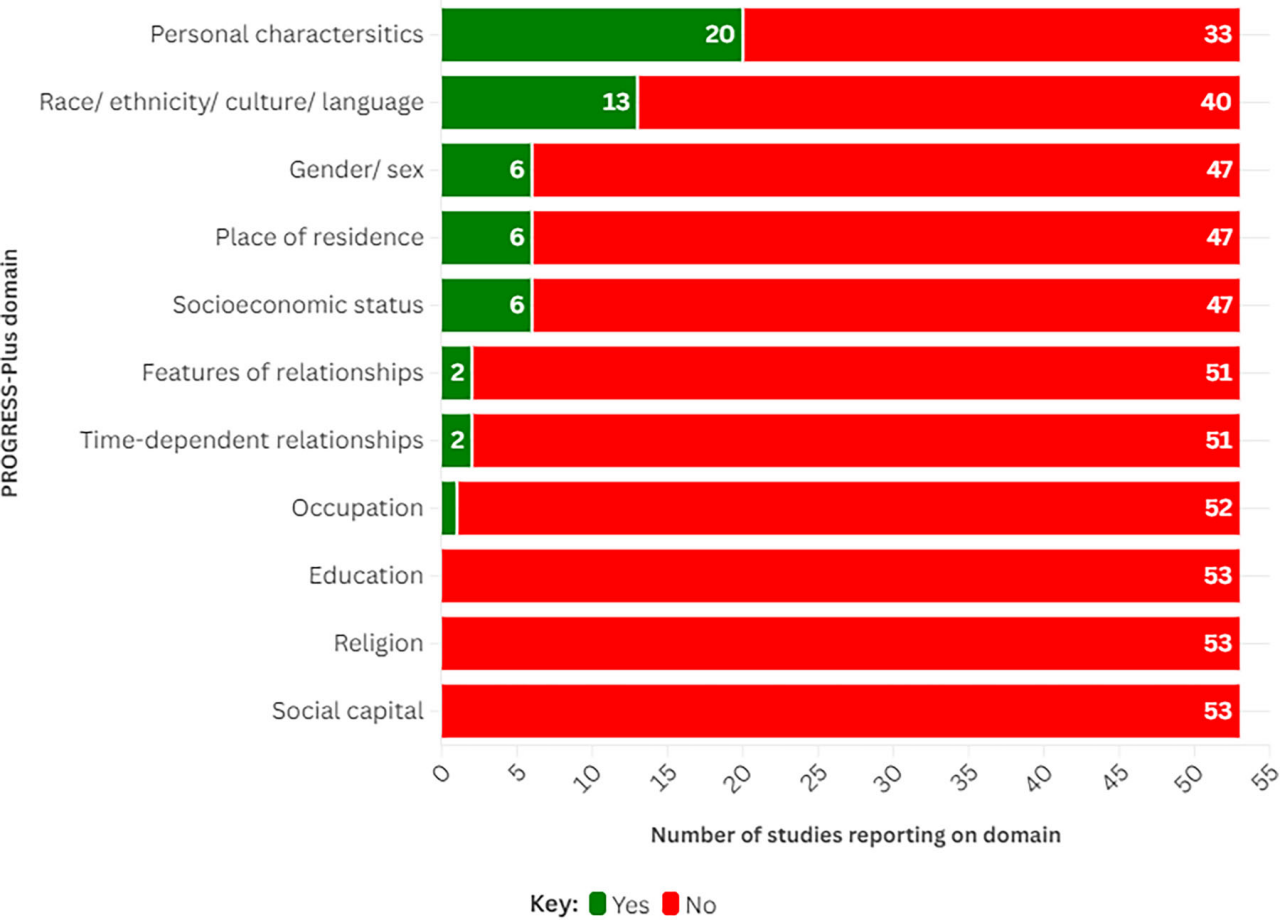
Frequency of integration of PROGRESS‐Plus characteristics in included frameworks.

As shown in Figure 3, there was limited reporting of specific equity considerations in terms of the PROGRESS‐Plus framework. Twenty of the 53 included frameworks suggested ways of involving people with specific personal characteristics, such as specific age groups or people with specific health conditions [24, 25, 28, 29, 30, 33, 40, 41, 42, 44, 48, 52, 53, 55, 56, 58, 60, 67, 70, 72], while 13 included considerations for how to involve people from different racial, ethnic, cultural backgrounds, or with first languages that differ from the researchers [23, 24, 33, 35, 36, 41, 42, 44, 45, 53, 58, 64, 68].

Six frameworks discussed considerations for involving those of differing gender/sex [24, 33, 44, 45, 46, 53, 58], place of residence [24, 42, 44, 53, 64, 68] and socioeconomic backgrounds [24, 33, 42, 44, 45, 46, 53]. Two frameworks each considered time‐dependent relationships (such as if someone has just left hospital, is in respite care or is at a temporary disadvantage) [53, 72], as well as features of relationships (such as smoking parents or if someone has been excluded from school) [30, 56]. Only one framework discussed the potential impact of occupation on involvement [23]. None of the included frameworks addressed the potential impact of education, religion, or social capital on involvement.

## 5 Discussion

### 5.1 Summary of Main Findings

In total, this rapid scoping review identified 55 reports of 53 eligible frameworks for embedding PPI in health and social care research. Thirty of these frameworks suggested they could be used in multiple forms of health and social care research. Twenty‐eight frameworks were developed in the UK. In general, reporting on approaches to recruit people to be involved in health and care research, how often they should be involved and their mode of involvement was limited. However, most frameworks suggested that people may have multiple and varied levels of involvement, from leading research to learning about its findings. Most frameworks reported a minimum of one approach to meeting at least one of the UK Standards for Public Involvement. However, details on approaches to involving groups with different characteristics influencing their ability to access and have good health, as defined by the PROGRESS‐Plus framework, were limited.

In their review of frameworks, Greenhalgh et al. [3] suggested that using ‘one size fits all’ approaches in frameworks for PPI may be less useful to researchers than resources that can be adapted and combined. This view was echoed by Chudyk et al. [6] in their scoping review of models and frameworks for patient engagement in health services research. Greenhalgh et al. [3] also noted that most of the published frameworks they identified had been seldom used beyond the groups that initially developed them. It is certainly true that any framework needs to be flexible and adapted to the specific context in which it is applied. This review found that most frameworks were neither targeted at involvement within specific types of health and care research (e.g., clinical trials, evidence synthesis, methodological research) nor aimed at involving specific groups.

However, this review also offers another possible alternative explanation for the phenomena Greenhalgh et al. described. By specifically examining how current frameworks provide detailed guidance on how to operationalise PPI in practice, this scoping review has demonstrated how many currently available frameworks perhaps do not provide the level of detail and guidance that researchers need to embed PPI into their work. As such, it is possible that the frameworks identified by this scoping review assume their end users have a higher overall level of knowledge and confidence in embedding PPI within health and social care research than is the case. This may particularly pose a challenge for researchers earlier in their career or with less experience of involving patients and members of the public in their work; it has already been previously noted that researchers can feel ‘ill equipped’ to undertake PPI [4].

Furthermore, there was a lack of consideration for diversity and equity across frameworks, as demonstrated by our assessment against the PROGRESS‐Plus domains. This finding aligns with a narrative review by Ocloo and Matthews [73], who highlighted uncertainties in how best to involve and support a diversity of individuals when undertaking PPI.

### 5.2 Strengths and Limitations of This Review

The conduct of this scoping review has numerous strengths. Firstly, we prospectively published the protocol on the Open Science Framework and transparently reported any deviations as part of this study [8]. The overall conduct was informed by guidance from the JBI and we reflected on the most recent guidance from the Cochrane Rapid Reviews Methods Group to robustly undertake the review within a short timeframe [7, 19, 74]. Furthermore, we included two members of the public in the research team (D.S. and B.H.), who assisted in governing the project and ensuring their perspectives were embedded into the work. Their involvement was guided by the principles outlined in the UK Standards for Public Involvement (see Supporting Information S2) [12].

However, the review was also potentially limited by some factors. As previously mentioned, there were some deviations from the pre‐published protocol, and this may have introduced an element of bias into the process. We also limited our searches to frameworks published from 2013 onwards. Although this was to capture frameworks that had been published since the NIHR carried out a review of existing work on principles and standards for public involvement in the National Health Service [12], this means potentially relevant frameworks published before this may have been excluded. Our definition of what constituted a framework for the purposes of this review focused on operational frameworks deemed to have practical guidance on how to implement a process or strategic imperative (see Table 1). This definition may have potentially limited the number of frameworks for PPI considered eligible for the scoping review. In particular, those which are more conceptual or evaluative in nature. Despite this, as we were most interested in identifying operational frameworks, this definition best fit our overall aim.

Furthermore, a single reviewer undertook screening and data charting of studies with a second checking a proportion of these for accuracy. Although our approach was informed by the latest guidance from the Cochrane Rapid Reviews Methods Group [74], it is possible that potentially relevant frameworks may have been erroneously excluded or there may be inaccuracies in the charting.

### 5.3 Implications for Research

This rapid scoping review has highlighted gaps in how current PPI frameworks in health and care research currently seem to lack requisite details to be able to allow researchers to embed PPI effectively and efficiently. This is problematic, given how previously mentioned work has highlighted how researchers sometimes feel they need more resources and guidance to conduct PPI [2, 4].

As such, any future frameworks for PPI in health and care research not only need to provide specific practical details, but also provide guidance to help embed PPI within specific health and care research methodologies. Although we found six frameworks specifically aimed at involvement in clinical trials [22, 29, 32, 40, 43, 54], most of the included frameworks provided broad advice not targeted at PPI within specific research methodologies (*N* = 30). As such, frameworks to aid researchers in embedding PPI in specific methodological disciplines are lacking. For example, we only identified one framework surrounding systematic reviews, which also provided guidance on embedding the views of other stakeholders alongside those of patients and the public [9].

Any new frameworks to aid researchers in embedding PPI within specific methodologies should be mindful that no research projects are identical, and that guidance may need to be flexible to meet the needs of end‐users and their individual research projects. As such, frameworks for PPI should aim to signpost to other resources that researchers could use to supplement their knowledge and the application of the tool, insofar as possible. This should, wherever possible, include signposting to relevant training resources to enable those at an earlier stage of their research career or with less experience of PPI to be able to build a foundation of knowledge on which to apply each framework. Given that Greenhalgh et al also noted that frameworks have seldom been used outside of the groups that developed them [3], developers of frameworks could also work together, such as in a community of practice, to ensure frameworks are evaluated and meet the needs of end users.

Furthermore, this review highlighted issues in how reported approaches to PPI in health and care research address inclusivity and diversity, as very few of the identified frameworks included considerations relating to PROGRESS‐Plus factors [10, 11]. This was particularly true of factors relating to a person's occupation, religion and social capital. A review by Ocloo et al into the theory, barriers and enablers to PPI across health and care research (2021) highlighted that there needs to be a wider group of individuals involved in health and care research, particularly from communities that have historically been marginalised or excluded [75]. Ocloo et al called for a targeted approach to involving specific groups, including those with low literacy, communication or language difficulties [75]. To do so, any future frameworks need to provide further guidance and consideration as to how research involvement can be inclusive and diverse. This could partly be achieved through greater cross‐talk between resources and guidance that already exists. For instance, frameworks could signpost to resources such as NIHR INCLUDE or the CHecklist for Inclusive COmmunity involvement in health research (CHICO) framework, which promotes inclusive approaches to involving community groups [42, 76]. Furthermore, frameworks could advocate for greater transparency in reporting who has been involved in research and how, thereby encouraging researchers to increase the visibility of diverse perspectives. By drawing on current literature and expertise within this area when developing any frameworks, and calling for enhanced transparency in reporting, strategies on how to include a more diverse range of people within health and care research may become more consistently reported and implemented.

## 6 Conclusion

Of the 53 frameworks we included in this review, most did not suggest specific methods surrounding how to operationalise PPI in health and care research. Although most frameworks suggested ways to meet at least one of the UK Standards for Public Involvement, such as communication and inclusive opportunities, very few suggested ways in which characteristics relating to equality, diversity and inclusion could be considered. As such, targeted frameworks are required to help provide clear guidance to researchers on how to involve people in a wider range of different types of health and social care research projects.

## Author Contributions

**Eugenie Evelynne Johnson:** conceptualisation, methodology, investigation, formal analysis, visualisation, funding acquisition, project administration, writing – original draft. **Sean Gill:** investigation, data curation, formal analysis, visualisation, writing – review and editing. **Madeleine Still:** investigation, data curation, data validation, writing – review and editing. **Daisy Trenchard:** investigation, data curation, validation, writing – review and editing. **Debbie Smith:** writing – review and editing. **Rebecca Harmston:** writing – review and editing. **Jane McDermott:** writing – review and editing. **Fiona Pearson:** conceptualisation, funding acquisition, writing – review and editing.

## Conflicts of Interest

The authors declare no conflicts of interest.

## Supporting information

PPI frameworks_supplementary material.

## Data Availability

The data that support the findings of this study are available from the corresponding author upon reasonable request.
