# Supplementary material for: Understanding the Scope, Intent and Extent of Published Conceptual Frameworks of Frameworks for Patient and Public Involvement in Health and Social Care Research: A Rapid Scoping Review
Source: Health Expect. 2025 Sep 4;28(5):e70425. doi: 10.1111/hex.70425 (PMC12411563; doi:10.1111/hex.70425)
Supplement: Supplementary file 1 — PPI frameworks_supplementary material. [file HEX-28-e70425-s001.docx]

**Supplementary Material 1: Differences between protocol and review**

The list below describes additions and amendments to the original protocol that occurred during the conduct of the scoping review.

**Data sources**

- We searched the National Co-ordinating Centre for Public Engagement website as an additional source for potential frameworks for inclusion.
- We undertook forward and backward citation chaining on relevant reviews to maximise scope of the searches and help ensure no relevant records were missed.

**Search strategies**

- The search strategy was not peer-reviewed by another information specialist as originally planned.

**Study selection**

- Two reviewers conducted the pilot screening of identified records from database searches instead of the planned three at full text.
- We clarified methods for selecting frameworks identified from sources such as websites; this was not detailed in the original protocol.

**Data charting**

- Three reviewers undertook pilot extraction for the data charting form instead of the planned two.

**Supplementary Material 2: Patient and public involvement according to the UK Standards for Public Involvement**

| **UK Standard** | **Summary of standard** | **Example activities in this work** |
| --- | --- | --- |
| Inclusive opportunities | Providing public involvement opportunities that are accessible and reach people according to research need | - Insofar as possible, ensuring that meetings are scheduled in a way that best suits the public research team members  - Flexible communication options (e.g. meetings, email) as required  - Choice and flexibility in how much to contribute to the governance of the work, as needed |
| Working together | Working together in a way that values contributions, builds and sustains mutual respect and productive relationships | - Ensuring the purpose of the role was clear and that expectations were set  - Involving members of the public in shaping and helping to interpret the scoping review, as well as reading and commenting on the manuscript  - Recognition of the role that the public research team members had on shaping the scoping review and how we interpreted the results |
| Support and learning | Offering and promoting support and learning opportunities to build confidence and skills for involvement in research | - Ensuring that the objectives of the work were clear and offering any ad hoc support and advice to the public research team members as required and desired |
| Communications | Using plain language for well-timed, relevant communications | - Flexible communication methods appropriate to the wishes of the public members (e.g. communication via email or meetings)  - Public members included in all research team correspondence, as appropriate, including notes and action points from all research team meetings |
| Impact | Identifying and sharing the difference public involvement makes to research | - Signposting of the contributions of the public research team members in the manuscript and as co-authors |
| Governance | Involving the public in the management, regulation, leadership and decision making within research | - Inclusion of the two public research team members in at least two research team meetings per month  - Roles of the public research team members discussed and updated throughout the work  - Resources in place to provide timely reimbursement for all time spent working on the project, according to NIHR recommended rates |
| Abbreviations: NIHR = National Institute for Health and Care Research; UK = United Kingdom Adapted from Crowe et al (2020)(6, 7) | | |

**Supplementary Material 3: Full eligibility criteria**

| **PCC domain** | **Included** | **Excluded** |
| --- | --- | --- |
| **Population** | - Any population: frameworks targeted at either the general population or specific population subgroups (e.g. children, people with specific conditions) | - No exclusion criteria |
| **Concept** | - Any framework to aid health and social care researchers to embed PPI into their work  - For the purposes of this review, a “framework” is any record that contained guiding questions or structures to enable researchers to conduct PPI in health and social care research  - Frameworks that provide guidance or an approach to operationalising involvement of patients and the public in research, according to the definitions provided by the NIHR  - Frameworks that provide guidance or an approach to operationalising involvement and engagement of patients and the public in research, according to the definitions provided by the NIHR | - Frameworks not aimed at health and social care research  - Frameworks only addressing engagement of patients and members of the public  - Frameworks for participation in qualitative or clinical research studies  - Guidance on how to report PPI within research |
| **Context** | - Any geographical context or country  - Any type of health or social care research (e.g. quantitative, qualitative and mixed-methods primary studies; and evidence syntheses, including systematic reviews of interventions, qualitative evidence syntheses, scoping reviews, mapping reviews or horizon scanning) | - Records published before 2013 |
| Abbreviations: NIHR = National Institute for Health and Care Research; PCC = population, concept, context; PPI = patient and public involvement | | |

**Supplementary Material 4: Search strategies**

**Database searches**

**PsycInfo**

1. exp Participation/ or exp Client Participation/ or exp Consumer Behavior/ or exp Consumer Attitudes/ or consumer participation.mp.

2. patient acceptance of health care.mp.

3. exp Health Attitudes/

4. consumer satisfaction.mp. or exp Consumer Satisfaction/

5. preference.mp. or exp Preferences/

6. (person-centred or Person Centered Psychotherapy).mp. [mp=title, book title, abstract, original title, name of substance word, subject heading word, floating sub-heading word, keyword heading word, organism supplementary concept word, protocol supplementary concept word, rare disease supplementary concept word, unique identifier, synonyms, population supplementary concept word, anatomy supplementary concept word]

7. Cooperative Behavior.mp. or Cooperation/

8. exp Self-Efficacy/

9. exp Adaptation/ or exp Adjustment/ or psychological adaptation.mp.

10. exp Coping Style/ or coping.mp. or exp Coping Behavior/

11. exp Self-Perception/ or Self-Concept/

12. Health Education/ or Mental Health Education/

13. Informed Consent/ or patient choice.mp. [mp=title, book title, abstract, original title, name of substance word, subject heading word, floating sub-heading word, keyword heading word, organism supplementary concept word, protocol supplementary concept word, rare disease supplementary concept word, unique identifier, synonyms, population supplementary concept word, anatomy supplementary concept word]

14. exp Decision Making/

15. exp Empowerment/

16. exp "Quality of Life"/

17. exp Autonomy/

18. exp Community Advocacy/ or exp Advocacy/ or exp Self-Advocacy/

19. exp Freedom/

20. exp Needs/ or exp Needs Assessment/

21. exp Support Groups/

22. (self help or self management).mp. [mp=title, book title, abstract, original title, name of substance word, subject heading word, floating sub-heading word, keyword heading word, organism supplementary concept word, protocol supplementary concept word, rare disease supplementary concept word, unique identifier, synonyms, population supplementary concept word, anatomy supplementary concept word]

23. exp Life Changes/ or exp Life Experiences/

24. "Attitude to Death".mp.

25. (expert patient or involvement or engagement or PPIE or "patient and public involvement and engagement").mp. [mp=title, book title, abstract, original title, name of substance word, subject heading word, floating sub-heading word, keyword heading word, organism supplementary concept word, protocol supplementary concept word, rare disease supplementary concept word, unique identifier, synonyms, population supplementary concept word, anatomy supplementary concept word]

26. exp Communication/

27. exp Emotions/ or exp Positive Emotions/ or exp Negative Emotions/ or exp Moral Emotions/ or emotion.mp.

28. exp Vignette Measure/ or vignette.mp.

29. 1 or 2 or 3 or 4 or 5 or 6 or 7 or 8 or 9 or 10 or 11 or 12 or 13 or 14 or 15 or 16 or 17 or 18 or 19 or 20 or 21 or 22 or 23 or 24 or 25 or 26 or 27 or 28

30. (framework or policy or guideline or guidance or guide or evaluation or protocol or consensus or method).mp. [mp=title, book title, abstract, original title, name of substance word, subject heading word, floating sub-heading word, keyword heading word, organism supplementary concept word, protocol supplementary concept word, rare disease supplementary concept word, unique identifier, synonyms, population supplementary concept word, anatomy supplementary concept word]

31. 29 and 30

32. limit 31 to yr="2013 -Current"

**Medline**

1. exp Consumer Participation/

2. ((patient$ or consumer$) adj3 (participat$ or decisi$ or decid$)).ti,ab.

3. "Patient Acceptance of Health Care"/

4. exp Attitude to Health/

5. consumer satisfaction/

6. exp "Consumer Satisfaction"/

7. Patient Preference/

8. "patient-focused".ti,ab.

9. "patient-centred".ti,ab.

10. "patient-centered".ti,ab.

11. (patient adj3 (attitude$ or preference$)).ti,ab.

12. "patient satisfaction".ti.

13. cooperative behavior/

14. exp self-efficacy/

15. self-efficacy.ti,ab.

16. exp adaptation, psychological/

17. coping.ti,ab.

18. ("self-perception" or "self-concept").ti,ab.

19. exp health education/

20. patient education as topic/

21. exp attitude to health/

22. health knowledge, attitudes, practice/

23. "informed choice".ti,ab.

24. "shared decision making".ti,ab.

25. empowerment.tw.

26. ("focus group" adj3 (patient$ or parent$ or famil$ or spouse$)).ti,ab.

27. "Quality of LIfe"/

28. "Quality of Life"/px

29. (QoL or "quality of life").ti.

30. personal autonomy/

31. self-concept/

32. Consumer Advocacy/

33. freedom/

34. needs assessment/

35. patient advocacy/

36. self-help groups/

37. life change events/

38. attitude to death/

39. patient-centered care/

40. exp professional-patient relations/

41. self-care/

42. self-management.ti.

43. ((patient$ or consumer$ or parent$ or famil$ or spouse$) adj (attitude$ or involvement or activation or view$ or preference$)).ti,ab.

44. "expert patient".ti,ab.

45. or/1-44

46. exp decision making/

47. exp communication/

48. stress,psychological/

49. emotions/

50. vignette.ti,ab.

51. or/46-50

52. exp Patients/px

53. (patient$ or consumer$).ti.

54. or/52-53

55. 51 and 54

56. "focus group$".ti,ab.

57. focus groups/

58. narration/

59. qualitative.ti.

60. or/56-59

61. 45 and 55 and 60

**Website searches**

*The King’s Fund (limited to 2013 onwards)*

| **Keyword searched** | **Total number of hits** | **Date searched** |
| --- | --- | --- |
| Framework | 402 | 18.7.24 |
| Guideline | 89 | 18.7.24 |
| Guidance | 288 | 18.7.24 |
| Evaluation | 129 | 18.7.24 |
| Policy | 1871 | 18.7.24 |

*NCCPE*

| **Keyword searched** | **Total number of hits** | **Date searched** |
| --- | --- | --- |
| Framework | 123 | 23.7.24 |
| Guideline | 19 | 23.7.24 |
| Guidance | 161 | 23.7.24 |
| Evaluation | 145 | 23.7.24 |
| Policy | 116 | 23.7.24 |

*NIHR Learning for involvement*

| **Keyword searched** | **Total number of hits** | **Date searched** |
| --- | --- | --- |
| Framework | 9 | 23.7.24 |
| Guideline | 4 | 23.7.24 |
| Guidance | 13 | 23.7.24 |
| Evaluation | 6 | 23.7.24 |
| Policy | 4 | 23.7.24 |

*Royal College of Midwifery (limited to 1.1.2013 onwards)*

| **Keyword searched** | **Total number of hits** | **Date searched** |
| --- | --- | --- |
| Framework | 81 | 23.7.24 |
| Guideline | 25 | 23.7.24 |
| Guidance | 935 | 23.7.24 |
| Evaluation | 67 | 23.7.24 |
| Policy | 357 | 23.7.24 |

*Royal College of Nursing*

| **Keyword searched** | **Total number of hits** | **Date searched** |
| --- | --- | --- |
| Framework | 227 | 23.7.24 |
| Guideline | 145 | 23.7.24 |
| Guidance | 631 | 23.7.24 |
| Evaluation | 111 | 23.7.24 |
| Policy | 665 | 23.7.24 |

*Royal College of Physicians*

| **Keyword searched** | **Total number of hits** | **Date searched** |
| --- | --- | --- |
| Framework | 210 | 18.7.24 |
| Guideline | 383 | 18.7.24 |
| Guidance | 577 | 18.7.24 |
| Evaluation | 288 | 18.7.24 |
| Policy | 650 | 18.7.24 |

**Supplementary Material 5: Data charting form**

| **Domain** | **Items** |
| --- | --- |
| Bibliographic details | - Reviewer initials - Checker initials - Source: database searching; web searching; citation chaining - Title - Authors (if applicable) - URL - Year published or updated - Type of source: journal article; webpage; report; etc. |
| Context | - Type of health/social care research - Country/countries developed - Brief details of the purpose of the framework - What part of the research process the PPIE relates to: study design; study conduct; dissemination/impact; multiple stages; other; not reported |
| Population | - Whether the framework is targeted at involving a general population or a specific subgroup of the population - If a specific population, details on what population the framework is targeted at |
| Modes of involvement adapted from the ACTIVE framework | - How PPI is proposed to be recruited: open – fixed; open – flexible; closed – invitation; closed – existing group; closed – purposive sampling; multiple; not reported - Proposed mode of involvement: one-time; continuous; both; not reported - Proposed method of involvement: direct interaction; no direct interaction; both direct and indirect interaction; not reported - If direct interaction, specific mode: face to face interaction; digital interaction (e.g. Zoom, Microsoft Teams); both; other; not reported - Proposed level of involvement: leading; controlling; contributing; receiving; multiple; not reported |
| Aspects of UK Standards for Public Involvement covered by the framework (all yes/no) | - Communication - Governance - Working together - Support and learning - Inclusive opportunities - Impact |
| PROGRESS-Plus domains featured (all yes/no) | - Place of residence - Race/ ethnicity/ culture/ language - Occupation - Gender/ sex - Religion - Education - Socioeconomic status - Social capital - Personal characteristics (e.g. age, disability) - Features of relationships (e.g. smoking parents, school exclusion) - Time-dependent relationships (e.g. leaving hospital, respite, temporary disadvantage) |
| Abbreviations: ACTIVE = Authors and Consumers Together Impacting on eVidencE; PPI = patient and public involvement; UK = United Kingdom; URL = uniform resource locator | |

**Supplementary Material 6: PRISMA-ScR checklist**

| **SECTION** | **ITEM** | **PRISMA-ScR CHECKLIST ITEM** | **REPORTED ON PAGE #** |
| --- | --- | --- | --- |
| **TITLE** | | | |
| Title | 1 | Identify the report as a scoping review. | Click here to enter text. |
| **ABSTRACT** | | | |
| Structured summary | 2 | Provide a structured summary that includes (as applicable): background, objectives, eligibility criteria, sources of evidence, charting methods, results, and conclusions that relate to the review questions and objectives. | Click here to enter text. |
| **INTRODUCTION** | | | |
| Rationale | 3 | Describe the rationale for the review in the context of what is already known. Explain why the review questions/objectives lend themselves to a scoping review approach. | Click here to enter text. |
| Objectives | 4 | Provide an explicit statement of the questions and objectives being addressed with reference to their key elements (e.g., population or participants, concepts, and context) or other relevant key elements used to conceptualize the review questions and/or objectives. | Click here to enter text. |
| **METHODS** | | | |
| Protocol and registration | 5 | Indicate whether a review protocol exists; state if and where it can be accessed (e.g., a Web address); and if available, provide registration information, including the registration number. | Click here to enter text. |
| Eligibility criteria | 6 | Specify characteristics of the sources of evidence used as eligibility criteria (e.g., years considered, language, and publication status), and provide a rationale. | Click here to enter text. |
| Information sources* | 7 | Describe all information sources in the search (e.g., databases with dates of coverage and contact with authors to identify additional sources), as well as the date the most recent search was executed. | Click here to enter text. |
| Search | 8 | Present the full electronic search strategy for at least 1 database, including any limits used, such that it could be repeated. | Click here to enter text. |
| Selection of sources of evidence† | 9 | State the process for selecting sources of evidence (i.e., screening and eligibility) included in the scoping review. | Click here to enter text. |
| Data charting process‡ | 10 | Describe the methods of charting data from the included sources of evidence (e.g., calibrated forms or forms that have been tested by the team before their use, and whether data charting was done independently or in duplicate) and any processes for obtaining and confirming data from investigators. | Click here to enter text. |
| Data items | 11 | List and define all variables for which data were sought and any assumptions and simplifications made. | Click here to enter text. |
| Critical appraisal of individual sources of evidence§ | 12 | If done, provide a rationale for conducting a critical appraisal of included sources of evidence; describe the methods used and how this information was used in any data synthesis (if appropriate). | Click here to enter text. |
| Synthesis of results | 13 | Describe the methods of handling and summarizing the data that were charted. | Click here to enter text. |
| **RESULTS** | | | |
| Selection of sources of evidence | 14 | Give numbers of sources of evidence screened, assessed for eligibility, and included in the review, with reasons for exclusions at each stage, ideally using a flow diagram. | Click here to enter text. |
| Characteristics of sources of evidence | 15 | For each source of evidence, present characteristics for which data were charted and provide the citations. | Click here to enter text. |
| Critical appraisal within sources of evidence | 16 | If done, present data on critical appraisal of included sources of evidence (see item 12). | Click here to enter text. |
| Results of individual sources of evidence | 17 | For each included source of evidence, present the relevant data that were charted that relate to the review questions and objectives. | Click here to enter text. |
| Synthesis of results | 18 | Summarize and/or present the charting results as they relate to the review questions and objectives. | Click here to enter text. |
| **DISCUSSION** | | | |
| Summary of evidence | 19 | Summarize the main results (including an overview of concepts, themes, and types of evidence available), link to the review questions and objectives, and consider the relevance to key groups. | Click here to enter text. |
| Limitations | 20 | Discuss the limitations of the scoping review process. | Click here to enter text. |
| Conclusions | 21 | Provide a general interpretation of the results with respect to the review questions and objectives, as well as potential implications and/or next steps. | Click here to enter text. |
| **FUNDING** | | | |
| Funding | 22 | Describe sources of funding for the included sources of evidence, as well as sources of funding for the scoping review. Describe the role of the funders of the scoping review. | Click here to enter text. |
| Abbreviations: JBI = Joanna Briggs Institute; PRISMA-ScR = Preferred Reporting Items for Systematic reviews and Meta-Analyses extension for Scoping Reviews. | | | |

**Supplementary Material 7: List of excluded studies**

*Exclusions from database searches*

| **Citation** | **Reason for exclusion** |
| --- | --- |
| Bailey S, Kalucy D, Nixon J, Williamson A, Wright D, Newman J, et al. Establishing an enduring co-production platform in Aboriginal health. Public health research & practice. 2022;32(2). | Ineligible concept |
| Banfield M, Randall R, O'Brien M, Hope S, Gulliver A, Forbes O, et al. Lived experience researchers partnering with consumers and carers to improve mental health research: Reflections from an Australian initiative. International journal of mental health nursing. 2018;27(4):1219-29. | Ineligible concept |
| Bisson M, Aubrey-Bassler K, Chouinard M-C, Doucet S, Ramsden VR, Dumont-Samson O, et al. Patient engagement in health implementation research: A logic model. Health expectations : an international journal of public participation in health care and health policy. 2023;26(5):1854-62. | Ineligible concept |
| Cacari-Stone L, Wallerstein N, Garcia AP, Minkler M. The promise of community-based participatory research for health equity: a conceptual model for bridging evidence with policy. American journal of public health. 2014;104(9):1615-23. | Ineligible concept |
| Carman KL, Dardess P, Maurer M, Sofaer S, Adams K, Bechtel C, et al. Patient and family engagement: a framework for understanding the elements and developing interventions and policies. Health affairs (Project Hope). 2013;32(2):223-31. | Ineligible concept |
| Carman KL, Workman TA. Engaging patients and consumers in research evidence: Applying the conceptual model of patient and family engagement. Patient education and counseling. 2017;100(1):25-9. | Ineligible concept |
| Darling E, Parra MA. Involving patients and the public in research. Nurse researcher. 2013;20(6):21-5. | Ineligible concept |
| DelNero P, McGregor A. From patients to partners. Science (New York, NY). 2017;358(6361):414. | Ineligible concept |
| Di Lorito C, Griffiths S, Poole M, Kaviraj C, Robertson M, Cutler N, et al. Patient and public involvement and engagement with underserved communities in dementia research: Reporting on a partnership to co-design a website for postdiagnostic dementia support. Health expectations : an international journal of public participation in health care and health policy. 2024;27(1):e13992. | Ineligible concept |
| Duhn L, Medves J. A 5-facet framework to describe patient engagement in patient safety. Health expectations : an international journal of public participation in health care and health policy. 2018;21(6):1122-33. | Ineligible concept |
| Duncanson E, Dansie K, Gutman T, Tong A, Howell M, Jesudason S, et al. 'Knowledge is power': A framework for partnering with consumers in developing and delivering a scientific meeting in nephrology. Nephrology (Carlton, Vic). 2020;25(5):379-83. | Ineligible concept |
| Edwards HA, Huang J, Jansky L, Mullins CD. What works when: mapping patient and stakeholder engagement methods along the ten-step continuum framework. Journal of comparative effectiveness research. 2021;10(12):999-1017. | Ineligible concept |
| Forsythe L, Heckert A, Margolis MK, Schrandt S, Frank L. Methods and impact of engagement in research, from theory to practice and back again: early findings from the Patient-Centered Outcomes Research Institute. Quality of life research : an international journal of quality of life aspects of treatment, care and rehabilitation. 2018;27(1):17-31. | Ineligible concept |
| Forsythe LP, Carman KL, Szydlowski V, Fayish L, Davidson L, Hickam DH, et al. Patient Engagement In Research: Early Findings From The Patient-Centered Outcomes Research Institute. Health affairs (Project Hope). 2019;38(3):359-67. | Ineligible concept |
| Gafari O, Bahrami-Hessari M, Norton J, Parmar R, Hudson M, Ndegwa L, et al. Building trust and increasing inclusion in public health research: co-produced strategies for engaging UK ethnic minority communities in research. Public health. 2024;233:90-9. | Ineligible concept |
| Gaffy E, Brijnath B, Dow B. Co-producing research with people impacted by dementia and service providers: issues and challenges. Public health research & practice. 2022;32(2). | Ineligible concept |
| Ganann R, McAiney C, Johnson W. Engaging older adults as partners in transitional care research. CMAJ : Canadian Medical Association journal = journal de l'Association medicale canadienne. 2018;190:S40-S1. | Ineligible concept |
| Gill SD, Gill M. Partnering with consumers: national standards and lessons from other countries. The Medical journal of Australia. 2015;203(3):134-6e.1. | Ineligible concept |
| Gittner LS. EMPOWERING PATIENTS TO BECOME BETTER PARTNERS. Journal of health and human services administration. 2015;38(2):276-88. | Ineligible concept |
| Goodwin E, Boddy K, Tatnell L, Hawton A. Involving Members of the Public in Health Economics Research: Insights from Selecting Health States for Valuation to Estimate Quality-Adjusted Life-Year (QALY) Weights. Applied health economics and health policy. 2018;16(2):187-94. | Ineligible concept |
| Greenhalgh T, Hinton L, Finlay T, Macfarlane A, Fahy N, Clyde B, et al. Frameworks for supporting patient and public involvement in research: Systematic review and co-design pilot. Health expectations : an international journal of public participation in health care and health policy. 2019;22(4):785-801. | Ineligible concept |
| Groot B, Abma T. Ethics framework for citizen science and public and patient participation in research. BMC medical ethics. 2022;23(1):23. | Ineligible concept |
| Grundy A, Keetharuth AD, Barber R, Carlton J, Connell J, Taylor Buck E, et al. Public involvement in health outcomes research: lessons learnt from the development of the recovering quality of life (ReQoL) measures. Health and quality of life outcomes. 2019;17(1):60. | Ineligible concept |
| Gutman T, Tong A, Howell M, Dansie K, Hawley CM, Craig JC, et al. Principles and strategies for involving patients in research in chronic kidney disease: report from national workshops. Nephrology, dialysis, transplantation : official publication of the European Dialysis and Transplant Association - European Renal Association. 2020;35(9):1585-94. | Ineligible concept |
| Headrick K, Thornton M, Hogan A, Deramore Denver B, Drake G, Wallen M. Consumer involvement in research - parent perceptions of partnership in cerebral palsy research: a qualitative study. Disability and rehabilitation. 2023;45(3):483-93. | Ineligible concept |
| Henry Akintobi T, Sheikhattari P, Shaffer E, Evans CL, Braun KL, Sy AU, et al. Community Engagement Practices at Research Centers in U.S. Minority Institutions: Priority Populations and Innovative Approaches to Advancing Health Disparities Research. International journal of environmental research and public health. 2021;18(12). | Ineligible concept |
| Hoekstra F, Mrklas KJ, Khan M, McKay RC, Vis-Dunbar M, Sibley KM, et al. A review of reviews on principles, strategies, outcomes and impacts of research partnerships approaches: a first step in synthesising the research partnership literature. Health research policy and systems. 2020;18(1):51. | Ineligible concept |
| Hoens AM, Belton J, Scott A, Ardern CL. Patients as Partners in Research: There Is Plenty of Help for Researchers. The Journal of orthopaedic and sports physical therapy. 2020;50(5):219-21. | Ineligible concept |
| Howe A. Principles of patient and public involvement in primary care research, applied to mental health research. A keynote paper from the EGPRN Autumn Conference 2017 in Dublin. The European journal of general practice. 2018;24(1):167-70. | Ineligible concept |
| Ismail MM, Gerrish K, Naisby A, Salway S, Chowbey P. Engaging minorities in researching sensitive health topics by using a participatory approach. Nurse researcher. 2014;22(2):44-8. | Ineligible concept |
| Jauffret-Roustide M, Granier J-M, Bertrand K. Penser paritairement les enjeux intimes et epistemologiques des recherches participatives L'exemple d'un compagnonnage pair-chercheur sur les politiques des drogues. 2024;35:79-84. | Ineligible concept |
| Jones M, Hoague D, Spriggs R, Catalan E, Adams N, Watkins T, et al. Establishing a Framework for Sustainable Community Action Research. Ethnicity & disease. 2022;32(4):333-40. | Ineligible concept |
| Jull J, Giles A, Graham ID. Community-based participatory research and integrated knowledge translation: advancing the co-creation of knowledge. Implementation science : IS. 2017;12(1):150. | Ineligible concept |
| Khodyakov D, Stockdale S, Jones A, Mango J, Jones F, Lizaola E. On measuring community participation in research. Health education & behavior : the official publication of the Society for Public Health Education. 2013;40(3):346-54. | Ineligible concept |
| Kirwan JR, de Wit M, Frank L, Haywood KL, Salek S, Brace-McDonnell S, et al. Emerging Guidelines for Patient Engagement in Research. Value in health : the journal of the International Society for Pharmacoeconomics and Outcomes Research. 2017;20(3):481-6. | Ineligible concept |
| Kirwan JR, de Wit MPT, Bingham CO, 3rd, Leong A, Richards P, Tugwell P, et al. Commentary: Patients as Partners: Building on the Experience of Outcome Measures in Rheumatology. Arthritis & rheumatology (Hoboken, NJ). 2016;68(6):1334-6. | Ineligible concept |
| Knowles SE, Allen D, Donnelly A, Flynn J, Gallacher K, Lewis A, et al. Participatory codesign of patient involvement in a Learning Health System: How can data-driven care be patient-driven care? Health expectations : an international journal of public participation in health care and health policy. 2022;25(1):103-15. | Ineligible concept |
| Lewis R, Boydell N, Blake C, Clarke Z, Kernaghan K, McMellon C. Involving young people in sexual health research and service improvement: conceptual analysis of patient and public involvement (PPI) in three projects. BMJ sexual & reproductive health. 2023;49(2):76-86. | Ineligible concept |
| Liabo K, Asare L, Ruthen P, Burton J, Staunton P, Day J. Emotion in public involvement: A conceptual review. Health Expectations. 2024;27(2):1-9. | Ineligible concept |
| Michaud S, Needham J, Sundquist S, Johnson D, Hanna S, Hosseinzadeh S, et al. Patient and Patient Group Engagement in Cancer Clinical Trials: A Stakeholder Charter. Current oncology (Toronto, Ont). 2021;28(2):1447-58. | Ineligible concept |
| Moser A, Korstjens I. Series: Practical guidance to qualitative research. Part 5: Co-creative qualitative approaches for emerging themes in primary care research: Experience-based co-design, user-centred design and community-based participatory research. The European journal of general practice. 2022;28(1):1-12. | Ineligible concept |
| Moule P, Davies R. A devolved model for public involvement in the field of mental health research: case study learning. Health expectations : an international journal of public participation in health care and health policy. 2016;19(6):1302-11. | Ineligible concept |
| Needham J, Taylor J, Nomikos D. Integrating Patient-Centred Research in the Canadian Cancer Trials Group. Current oncology (Toronto, Ont). 2021;28(1):630-9. | Ineligible concept |
| Nixon J. Partnering with Consumers - Why would I do it? What is it? How do I do it? Journal of medical radiation sciences. 2024;71(2):170-3. | Ineligible concept |
| Odii A, Akamike IC, Mbachu CO, Onwujekwe O. Factors influencing adoption of sexual and reproductive health intervention for adolescents in Ebonyi, Nigeria. BMC health services research. 2024;24(1):643. | Ineligible concept |
| Oehrlein EM, Graff JS, Harris J, Perfetto EM. Patient-Community Perspectives on Real-World Evidence: Enhancing Engagement, Understanding, and Trust. The patient. 2019;12(4):375-81. | Ineligible concept |
| Ogenchuk M, Graham J, Uswak G, Graham H, Weiler R, Ramsden VR. Pediatric oral health: community-based participatory research. BMC pediatrics. 2022;22(1):93. | Ineligible concept |
| Olagundoye O, Gibson W, Wagg A. A protocol for the co-creation and usability/acceptability testing of an evidence-based, patient-centred intervention for self-management of urinary incontinence in older men. PloS one. 2024;19(8):e0306080. | Ineligible concept |
| Olding M, Hayashi K, Pearce L, Bingham B, Buchholz M, Gregg D, et al. Developing a patient-reported experience questionnaire with and for people who use drugs: A community engagement process in Vancouver's Downtown Eastside. International Journal of Drug Policy. 2018;59:16-23. | Ineligible concept |
| Omale UI, Ogbonnaya LU, Iyare O, Nnachi OO. System-wide governance challenges of the Ebonyi State Malaria Elimination Programme and recommendations for malaria health system strengthening: a qualitative study among stakeholders in Ebonyi state, Nigeria. BMJ open. 2024;14(5):e082598. | Ineligible concept |
| Pinsoneault LT, Connors ER, Jacobs EA, Broeckling J. Go Slow to Go Fast: Successful Engagement Strategies for Patient-Centered, Multi-Site Research, Involving Academic and Community-Based Organizations. Journal of general internal medicine. 2019;34(1):125-31. | Ineligible concept |
| Pomey M-P, Bush PL, Demers-Payette O, L'Esperance A, Lochhead L, Ganache I, et al. Developing recommendations for the diagnosis and treatment of Lyme disease: the role of the patient's perspective in a controversial environment. International journal of technology assessment in health care. 2020;37:e11. | Ineligible concept |
| Rocque R, Chipenda Dansokho S, Grad R, Witteman HO. What matters to patients and families: A content and process framework for clarifying preferences, concerns, and values. Medical Decision Making. 2020;40(6):722-34. | Ineligible concept |
| Rogers S, Howard G. Patient and community engagement: the approach at university health network. Healthcare quarterly (Toronto, Ont). 2014;17(3):70-3. | Ineligible concept |
| Sheridan S, Schrandt S, Forsythe L, Hilliard TS, Paez KA. The PCORI Engagement Rubric: Promising Practices for Partnering in Research. Annals of family medicine. 2017;15(2):165-70. | Ineligible concept |
| Silberberg M, Martinez-Bianchi V. Community and Stakeholder Engagement. Primary care. 2019;46(4):587-94. | Ineligible concept |
| Smith KL, Main E, Bauer ME. Moving from Principle to Practice: A Researcher's Guide to Co-Leading Engaged Research with Community Partners and Patients with Lived Experience to Reduce Maternal Mortality and Morbidity for Maternal Sepsis. Maternal and child health journal. 2024;28(8):1315-23. | Ineligible concept |
| Thomson A, Peasgood E, Robertson S. The Youth Patient and Public Involvement Cafe-A youth-led model for meaningful involvement with children and young people. Health expectations : an international journal of public participation in health care and health policy. 2022;25(6):2893-901. | Ineligible concept |

*Exclusions from citation chaining*

| **Citation** | **Reason for exclusion** |
| --- | --- |
| Abelson J, Tripp L, MacNeil M, Lang A, Fancott C, Ganann R, Granieri M, Hofstetter C, King B, Kristy BL, Maybee A. Development of the Engage with Impact Toolkit: A comprehensive resource to support the evaluation of patient, family and caregiver engagement in health systems. Health Expectations. 2023 Jun;26(3):1255-65. | Ineligible concept |
| Abelson J. Patient engagement in health technology assessment: What constitutes “meaningful” and how we might get there. J Health Serv Res Policy. 2018;23(2):69–71. | Ineligible concept |
| Aboaja, A., Forsyth, B., Bates, H., & Wood, R. (2021). Involving service users to identify research priorities in a UK forensic mental health service. BJPsych Bulletin, 45(6), 321–326. https://doi.org/10.1192/bjb.2020.131 | Ineligible concept |
| Aceves-Martins M, Aleman-Diaz AY, Giralt M, Solà R. Involving young people in health promotion, research and policy-making: practical recommendations. Int J Qual Health Care. 2019; 31(2): 147-153. | Ineligible concept |
| Arnstein L, Wadsworth AC, Yamamoto BA, Stephens R, Sehmi K, Jones R, et al. Patient involvement in preparing health research peer-reviewed publications or results summaries: a systematic review and evidence-based recommendations. Res Involv Engagem. (2020) 6:1–14. doi: 10.1186/s40900-020-00190-w | Ineligible concept |
| Baines R, Bradwell H, Edwards K, Stevens S, Prime S, Tredinnick‐Rowe J, Sibley M, Chatterjee A. Meaningful patient and public involvement in digital health innovation, implementation and evaluation: a systematic review. Health Expectations. 2022 Aug;25(4):1232-45. | Ineligible concept |
| Baquet CR, Bromwell JL, Hall MB, Frego JF. Rural community-academic partnership model for community engagement and partnered research. Prog Community Health Partnersh. 2013;7(3):281–90. | Ineligible concept |
| Barnieh L, Jun M, Laupacis A, et al. Determining research priorities through partnership with patients: an overview. Semin Dial 2015;28:141–6. | Ineligible concept |
| Belone L, Lucero JE, Duran B, et al. Community-based participatory research conceptual model: Community partner consultation and face validity. Qual Health Res. 2016; 26(1): 117-135. | Ineligible concept |
| Berwick A, Holland G, Power B, Rebane A, Butler B, Orsi NM. Patient and public involvement (PPI) in computer-aided diagnostics in digital histopathology. Diagnostic Histopathology. 2023 Jun 27. | Ineligible concept |
| Boaz A, Hanney S, Borst R, O'Shea A, Kok M. How to engage stakeholders in research: design principles to support improvement. Health Res Policy Syst. 2018;16(1):60. | Ineligible concept |
| Bobbio L. Designing effective public participation. Policy Soc. (2019) 38:41–57. doi: 10.1080/14494035.2018.1511193 | Ineligible concept |
| Boivin A, L’Espérance A, Gauvin FP, et al. Patient and public engagement in research and health system decision making: a systematic review of evaluation tools. Health Expect. 2018;21(6):1075–84. | Ineligible study design |
| Boursaw B, Oetzel JG, Dickson E, Thein TS, Sanchez‐Youngman S, Peña J, Parker M, Magarati M, Littledeer L, Duran B, Wallerstein N. Scales of practices and outcomes for community‐engaged research. American journal of community psychology. 2021 Jun;67(3-4):256-70. | Ineligible concept |
| Burton L, Ní Shé É, Olliver S. Embedding an empowerment evaluation framework to create a ‘win-win’ engaged research partnership with communities. Austral J Univ Commun Engagem. 2015; 10(2): 63-81. | Ineligible concept |
| Cancer Research UK. Patient involvement toolkit for researchers. <https://www.cancerresearchuk.org/funding-for-researchers/patient-involvement-toolkit-for-researchers> | Ineligible concept |
| Capobianco L, Faija C, Cooper B, Brown L, McPhillips R, Shields G, Wells A. A framework for implementing Patient and Public Involvement in mental health research: The PATHWAY research programme benchmarked against NIHR standards. Health Expectations. 2023 Apr;26(2):640-50. | Ineligible concept |
| Carroll P, Smith É, Dervan A, McCarthy C, Woods I, Beirne C, Harte G, O'Flynn D, Quinlan J, O'Brien FJ, Flood M. The Development of Principles for Patient and Public Involvement (PPI) in Preclinical Spinal Cord Research: A Modified Delphi Study. Health Expectations. 2024 Aug;27(4):e14130. | Ineligible concept |
| Ceasar J, Peters-Lawrence MH, Mitchell V, Powell-Wiley TM. The communication, awareness, relationships and empowerment (CARE) model: an effective tool for engaging urban communities in community-based participatory research. Int J Environ Res Public Health. 2017; 14(11): 1422. | Ineligible concept |
| Center for Commununity Health and Development at the University of Kansas. Community Toolbox. Section 8: Identifying and analyzing stakeholders and their interests. 2016 Available from: https://ctb.ku.edu/en/table-of-contents/participation/encouraging-involvement/identify-stakeholders/main. | Ineligible concept |
| Centre of Excellence on Partnership with Patients and the Public (CEPPP). Patient and public engagement evaluation toolkit. 2021. https://ceppp.ca/en/evaluation-toolkit/ | Ineligible concept |
| Chauhan A, Leefe J, Shé ÉN, Harrison R. Optimising co-design with ethnic minority consumers. International journal for equity in health. 2021 Dec;20:1-6. | Ineligible concept |
| Cheng VWS, Piper SE, Ottavio A, Davenport TA, Hickie IB. Recommendations for designing health information technologies for mental health drawn from self-determination theory and co-design with culturally diverse populations: template analysis. J Med Internet Res. 2021; 23(2):e23502. doi:10.2196/23502 | Ineligible concept |
| Chudyk AM, Horrill T, Waldman C, Demczuk L, Shimmin C, Stoddard R, Hickes S, Schultz AS. Scoping review of models and frameworks of patient engagement in health services research. BMJ open. 2022 Aug 1;12(8):e063507. | Ineligible study design |
| Cleemput I, Dauvrin M, Kohn L, Mistiaen P, Christiaens W, Léonard C. Position of KCE on Patient Involvement in Health Care Policy Research. Belgian Health Care Knowledge Centre (KCE); 2019. KCE Reports 320 D/2019/10273/57. <https://kce.fgov.be/en/position-of-kce-on-patient-involvement-in-health-care-policy-research> | Ineligible concept |
| Cluley V, Ziemann A, Feeley C, Olander EK, Shamah S, Stavropoulou C. Mapping the role of patient and public involvement during the different stages of healthcare innovation: a scoping review. Health Expectations. 2022 Jun;25(3):840-55. | Ineligible concept |
| Collins M, Long R, Page A, Popay J, Lobban F. Using the Public Involvement Impact Assessment Framework to assess the impact of public involvement in a mental health research context: a reflective case study. Health Expect. 2018; 21(6): 950-963. | Ineligible concept |
| Colomer‐Lahiguera S, Steimer M, Ellis U, Eicher M, Tompson M, Corbière T, Haase KR. Patient and public involvement in cancer research: A scoping review. Cancer Medicine. 2023 Jul;12(14):15530-43. | Ineligible concept |
| Cook N, Siddiqi N, Twiddy M, Kenyon R. Patient and public involvement in health research in low and middle-income countries: a systematic review. BMJ open. 2019 May 1;9(5):e026514. | Ineligible concept |
| Corbie-Smith G, Wynn M, Richmond A, et al. Stakeholder-driven, consensus development methods to design an ethical framework and guidelines for engaged research. PloS One. 2018; 13(6): e0199451. | Ineligible concept |
| Costello W, Dorris E. Laying the groundwork: Building relationships for public and patient involvement in pre‐clinical paediatric research. Health Expectations. 2020 Feb;23(1):96-105. | Ineligible concept |
| Couch J, Durant B, Hill J. Uncovering marginalised knowledges: undertaking research with hard-to-reach young people. Int J Mult Res Approaches. 2014; 8(1): 15-23. | Ineligible concept |
| Coupe N, Mathieson A. Patient and public involvement in doctoral research: impact, resources and recommendations. Health Expectations. 2020 Feb;23(1):125-36. | Ineligible concept |
| Creating a space for young people's involvement in health research. 2018. https://generationr.org.uk/wp-content/uploads/2018/06/Health-Research-Authority-Staff-Day26April18.pdf | Ineligible concept |
| Davis, R. E., Jacklin, R., Sevdalis, N., & Vincent, C. A. (2007). Patient involvement in patient safety: What factors influence patient participation and engagement? Health Expectations, 10(3), 259–267. <https://doi.org/10.1111/j.1369-7625.2007.00450.x> | Ineligible concept |
| Dawson S, Campbell SM, Giles SJ, et al. Black and minority ethnic group involvement in health and social care research: a systematic review. Health Expect. 2018; 21(1): 3-22. | Ineligible concept |
| Dengsø KE, Lindholm ST, Herling SF, Pedersen M, Nørskov KH, Collet MO, Nielsen IH, Christiansen MG, Engedal MS, Moen HW, Piil K. Patient and public involvement in Nordic healthcare research: a scoping review of contemporary practice. Research Involvement and Engagement. 2023 Aug 30;9(1):72. | Ineligible study design |
| Deverka PA, Lavallee DC, Desai PJ, et al. Stakeholder participation in comparative effectiveness research: defining a framework for effective engagement. J Comp Eff Res 2012; 1: 181–94. doi:10.2217/cer.12.7 | Published pre-2013 |
| Ekezie W, Routen A, Denegri S, Khunti K. Patient and public involvement for ethnic minority research: an urgent need for improvement. Journal of the Royal Society of Medicine. 2021 Jul;114(7):347-50. | Ineligible concept |
| Evans BA, Porter A, Snooks H, Burholt V. A co-produced method to involve service users in research: the SUCCESS model. BMC Med Res Methodol. 2019;19(1):34. | Ineligible concept |
| Faulkner A, NSUN National Involvement Team. Involvement for Influence. 4Pi National Involvement Partnership for Mental Health; 2016. http://www.nsun.org.uk/assets/downloadableFiles/4PiNationalInvolvementStandardsFullReport20152.pdf. | Ineligible concept |
| Feldman D, Kruger P, Delbecque L, Duenas A, Bernard-Poenaru O, Wollenschneider S, et al. Co-creation of practical “how-to guides” for patient engagement in key phases of medicines development—from theory to implementation. Res Involv Engagem. (2021) 7:1–11. doi: 10.1186/s40900-021-00294-x | Ineligible study design |
| Finderup J, Buur LE, Tscherning SC, et al. Developing and testing guidance to support researchers engaging patient partners in health-related research. Res Involv Engagem. 2022; 8(1): 1-11. | Ineligible concept |
| Finley N, Swartz TH, Cao K, Tucker JD. How to make your research jump off the page: co-creation to broaden public engagement in medical research. PLoS Medicine. 2020 Sep 14;17(9):e1003246. | Ineligible study design |
| Fleurence RL, Forsythe LP, Lauer M, Rotter J, Ioannidis JPA, Beal A, et al. Engaging patients and stakeholders in research proposal review: the patient-centered outcomes research institute. Ann Int Med. (2014) 161:122–30. doi: 10.7326/M13-2412 | Ineligible concept |
| Flinders, M., Wood, M., & Cunningham, M. (2016). The politics of co-production: Risks, limits and pollution. Evidence & Policy, 12(2), 261–279. <https://doi.org/10.1332/174426415X14412037949967> | Ineligible study design |
| Foley K, Lunnay B, Kevin C, Ward PR. Developing a Women's Thought Collective methodology for health research: The roles and responsibilities of researchers in the reflexive co‐production of knowledge. Health Expectations. 2023 Oct;26(5):1954-64. | Ineligible study design |
| Forsythe LP, Szydlowski V, Murad MH, et al. A systematic review of approaches for engaging patients for research on rare diseases. J Gen Intern Med. 2014;29(Suppl 3):S788-800. | Ineligible study design |
| Frampton SB, Guastello S, Hoy L, Naylor M, Sheridan S, Johnston-Fleece M. Harnessing Evidence and Experience to Change Culture: A Guiding Framework for Patient and Family Engaged Care. National Academy of Medicine. 2017; https://nam.edu/wp-content/uploads/2017/01/Harnessing-Evidence-andExperience-to-Change-Culture-A-GuidingFramework-for-Patient-and-Family-Engaged-Care.pdf. | Ineligible concept |
| Frank L, Forsythe L, Ellis L, et al. Conceptual and practical foundations of patient engagement in research at the patient-centered outcomes research Institute. Qual Life Res 2015;24:1033–41.doi:10.1007/s11136-014-0893-3 | Ineligible concept |
| Fruytier SE, Vat LE, Camp R, Houÿez F, De Keyser H, Dunne D, Marchi D, McKeaveney L, Pitt RH, Pittens CA, Vaughn MF. Monitoring and evaluation of patient engagement in health product research and development: co-creating a framework for community advisory boards. Journal of Patient-Centered Research and Reviews. 2022;9(1):46. | Ineligible concept |
| Gaasterland CM, Jansen-van der Weide MC, Vroom E, Leeson-Beevers K, Kaatee M, Kaczmarek R, Bartels B, van der Pol WL, Roes KC, van der Lee JH. The POWER-tool: recommendations for involving patient representatives in choosing relevant outcome measures during rare disease clinical trial design. Health Policy. 2018 Dec 1;122(12):1287-94. | Ineligible concept |
| Galvin M, Kennan A, Shé ÉN. A design-led framework for engaged research: Using a design approach to understand and place the public at the core of health and social care. Administration. 2021;69(3):1-8. | Ineligible study design |
| Garcia-Iglesias J, Beange I, Davidson D, Goopy S, Huang H, Murray F, Porteous C, Stevenson E, Rhodes S, Watson F, Fletcher-Watson S. Ethical considerations in public engagement: developing tools for assessing the boundaries of research and involvement. Research Involvement and Engagement. 2024 Dec;10(1):1-8. | Ineligible concept |
| Garratt A, Sagen J, Børøsund E, Varsi C, Kjeken I, Dagfinrud H, Moe RH. The public and patient engagement evaluation tool: forward-backwards translation and cultural adaption to Norwegian. BMC musculoskeletal disorders. 2022 Jun 9;23(1):556. | Ineligible study design |
| Gesell SB, Klein KP, Halladay J, et al. Methods guiding stakeholder engagement in planning a pragmatic study on changing stroke systems of care. J Clin Transl Sci 2017;1:121–8.doi:10.1017/cts.2016.26 | Ineligible study design |
| Gibbins KJ, Lo JO. What matters to whom: patient and public involvement in research. Clinical obstetrics and gynecology. 2022 Jun 1;65(2):268-76. | Ineligible study design |
| Gibson A, Welsman J, Britten N. Evaluating patient and public involvement in health research: from theoretical model to practical workshop. Health Expect 2017;20:826–35.doi:10.1111/hex.12486 | Duplicate of a record found while handsearching |
| Goldsmith LP, Morshead R, McWilliam C, Forbes G, Ussher M, Simpson A, Lucock M, Gillard S. Co-producing randomized controlled trials: How do we work together?. Frontiers in Sociology. 2019 Mar 29;4:21. | Ineligible study design |
| Green D, Bryant V, Edwards S, Kemp C, McKenzie M, Shah S, Soulsby I. Then there were seven: a commentary on creating a public involvement strategy group for a policy research unit in behavioural science. Research Involvement and Engagement. 2023 Feb 4;9(1):1. | Ineligible study design |
| Hall AE, Bryant J, Sanson‐Fisher RW, Fradgley EA, Proietto AM, Roos I. Consumer input into health care: time for a new active and comprehensive model of consumer involvement. Health Expectations. 2018 Aug;21(4):707-13. | Ineligible study design |
| Hamakawa N, Nakano R, Kogetsu A, Coathup V, Kaye J, Yamamoto BA, et al. Landscape of participant-centric initiatives for medical research in the United States, the United Kingdom, and Japan: scoping review. J Med Internet Res. (2020) 22:1–17. doi: 10.2196/16441 | Ineligible study design |
| Hawke LD, Relihan J, Miller J, et al. Engaging youth in research planning, design and execution: practical recommendations for researchers. Health Expect. 2018; 21(6): 944-949. | Ineligible concept |
| Health Research Authority (HRA). (2017). UK policy framework for health and social care research. Retrieved June 5, 2023, from https://www.hra.nhs.uk/planning-and-improving-research/policies-standards-legislation/uk-policy-framework-health-social-care-research/uk-policy-framework-health-and-social-care-research/ | Ineligible concept |
| Heaven A, Brown L, Foster M, Clegg A. Keeping it credible in cohort multiple Randomised Controlled Trials: the Community Ageing Research 75+(CARE 75+) study model of patient and public involvement and engagement. Res Involv Engagem. 2016; 2(1): 30. | Ineligible concept |
| Hemming, L., Pratt, D., Bhatti, P. P., Shaw, J., & Haddock, G. (2021). Involving an individual with lived-experience in a co-analysis of qualitative data. Wiley. <https://doi.org/10.1111/hex.13188> | Ineligible concept |
| Holroyd-Leduc J, Resin J, Ashley L. Giving voice to older adults living with frailty and their family caregivers: engagement of older adults living with frailty in research, health care decision making, and in health policy. Res Involv Engagem. 2016; 17(2): 23. | Ineligible study design |
| Horobin A. Going the extra mile–creating a co-operative model for supporting patient and public involvement in research. Res Involv Engagem. 2016; 2(1): 9. | Ineligible study design |
| Huddy JR, Weldon SM, Ralhan S, et al. Sequential simulation (SqS) of clinical pathways: a tool for public and patient engagement in point-of-care diagnostics. BMJ Open. 2016; 6(9):e011043. doi:10.1136/bmjopen-2016-011043 | Ineligible concept |
| Iwarsson S, Edberg AK, Ivanoff SD, Hanson E, Jönson H, Schmidt S. Understanding user involvement in research in aging and health. Gerontology and geriatric medicine. 2019 Dec;5:2333721419897781. | Ineligible concept |
| Jackson T, Pinnock H, Liew SM, Horne E, Ehrlich E, Fulton O, Worth A, Sheikh A, De Simoni A. Patient and public involvement in research: from tokenistic box ticking to valued team members. BMC medicine. 2020 Dec;18:1-7. | Ineligible concept |
| Javanparast, S., Robinson, S., Kitson, A., & Arciuli, J. (2022). Embedding research codesign knowledge and practice: Learnings from researchers in a new research institute in Australia. Springer Science and Business Media LLC. <https://doi.org/10.1186/s40900-022-00392-4> | Ineligible concept |
| Jayes M, Moulam L, Meredith S, Whittle H, Lynch Y, Goldbart J, Judge S, Webb E, Meads D, Hemsley B, Murray J. Making public involvement in research more inclusive of people with complex speech and motor disorders: the I-ASC Project. Qualitative Health Research. 2021 Jun;31(7):1260-74. | Ineligible concept |
| Jull J, Giles A, Boyer Y, Stacey D, Lodge M. Development of a collaborative research framework: an example of a study conducted by and with a First Nations, Inuit and Métis Women’s community and its research partners. ACME Int J Crit Geogr. 2018;17(3):671–86. | Ineligible concept |
| Kaisler RE, Kulnik ST, Klager E, Kletecka-Pulker M, Schaden E, Stainer-Hochgatterer A. Introducing patient and public involvement practices to healthcare research in Austria: strategies to promote change at multiple levels. BMJ open. 2021 Aug 1;11(8):e045618. | Ineligible concept |
| Karazivan P, Dumez V, Flora L, et al. The patient-as-partner approach in health care: a conceptual framework for a necessary transition. Acad Med. 2015;90(4):437–41. | Ineligible concept |
| Katirai A, Kogetsu A, Kato K, Yamamoto B. Patient involvement in priority-setting for medical research: a mini review of initiatives in the rare disease field. Frontiers in Public Health. 2022 Jul 19;10:915438. | Ineligible concept |
| Kauffman KS, Dosreis S, Ross M, Barnet B, Onukwugha E, Mullins CD. Engaging hard-to-reach patients in patient-centered outcomes research. J Comp Eff Res. 2013; 2(3): 313-324. | Ineligible concept |
| Kearney A, Williamson P, Young B, Bagley H, Gamble C, Denegri S, Muir D, Simon NA, Thomas S, Elliot JT, Bulbeck H. Priorities for methodological research on patient and public involvement in clinical trials: A modified Delphi process. Health Expectations. 2017 Dec;20(6):1401-10. | Ineligible concept |
| Kelly BS, Kirwan A, Quinn MS, Kelly AM, Mathur P, Lawlor A, Killeen RP. The ethical matrix as a method for involving people living with disease and the wider public (PPI) in near-term artificial intelligence research. Radiography. 2023 May 1;29:S103-11. | Ineligible concept |
| Kerr C, McConnell K, Savage H, Acheson M. Implementing public involvement standards in cerebral palsy register research. Frontiers in Rehabilitation Sciences. 2022 Nov 17;3:903167. | Ineligible concept |
| Kogetsu A, Kato K. Framework and practical guidance for the ethical use of electronic methods for communication with participants in medical research. Journal of Medical Internet Research. 2022 Apr 20;24(4):e33167. | Ineligible concept |
| Li KK, Abelson J, Giacomini M, Contandriopoulos D. Conceptualizing the use of public involvement in health policy decision-making. Social Science & Medicine. 2015 Aug 1;138:14-21. | Ineligible concept |
| Lignou S, Sheehan M, Singh I. ‘A commitment to Equality, Diversity and Inclusion’: a conceptual framework for equality of opportunity in Patient and Public Involvement in research. Research Ethics. 2024 Apr;20(2):288-303. | Ineligible concept |
| Luna Puerta L, Smith HE. The “PPI Hawker”: an innovative method for patient and public involvement (PPI) in health research. Research Involvement and Engagement. 2020 Dec;6:1-2. | Ineligible concept |
| Makey LM, Walsh CL, Salih I. Co-production: what it is and how it can ensure inclusive practice for service users and staff. Nursing management. 2023 Oct 5;30(5). | Ineligible concept |
| Manafo E, Petermann L, Mason-Lai P, Vandall-Walker V. Patient engagement in Canada: a scoping review of the ‘how’and ‘what’of patient engagement in health research. Health research policy and systems. 2018 Dec;16:1-1. | Ineligible concept |
| McLean RK, Carden F, Aiken AB, Armstrong R, Bray J, Cassidy CE, Daub O, Di Ruggiero E, Fierro LA, Gagnon M, Hutchinson AM. Evaluating the quality of research co-production: research quality plus for co-production (RQ+ 4 co-pro). Health Research Policy and Systems. 2023 Jun 13;21(1):51. | Duplicate of record from database searches |
| McParland C, Johnston B, Alassoud B, Drummond M, Farnood A, Purba CI, Seckin M, Thanthong S. Involving patients and the public in nursing PhD projects: practical guidance, potential benefits and points to consider. Nurse Researcher. 2024 Mar 13;32(1). | Ineligible concept |
| Menzies JC, Morris KP, Duncan HP, Marriott JF. Patient and public involvement in Paediatric Intensive Care research: considerations, challenges and facilitating factors. Research Involvement and Engagement. 2016 Dec;2:1-6. | Ineligible concept |
| Miah J, Sheikh S, Francis RC, et al. Patient and public involvement for dementia research in low- and middle-income countries: developing capacity and capability in South Asia. Front Neurol. 2021; 12:637000. doi:10.3389/fneur.2021.637000 | Ineligible concept |
| Morris AC, Douch S, Popnikolova T, McGinley C, Matcham F, Sonuga-Barke E, Downs J. A framework for remotely enabled co-design with young people: its development and application with neurodiverse children and their caregivers. Frontiers in Psychiatry. 2024 Aug 16;15:1432620. | Ineligible concept |
| Mortimer D, Iezzi A, Dickins M, Johnstone G, Lowthian J, Enticott J, Ogrin R. Using co‐creation and multi‐criteria decision analysis to close service gaps for underserved populations. Health Expectations. 2019 Oct;22(5):1058-68. | Ineligible concept |
| Mrklas KJ, Boyd JM, Shergill S, Merali S, Khan M, Nowell L, Goertzen A, Pfadenhauer LM, Paul K, Sibley KM, Swain L. Tools for assessing health research partnership outcomes and impacts: a systematic review. Health Research Policy and Systems. 2023 Jan 5;21(1):3. | Ineligible concept |
| Musesengwa R, Chimbari MJ, Mukaratirwa S. A framework for community and stakeholder engagement: experiences from a multicenter study in Southern Africa. J Empir Res Hum Res Ethics. 2018; 13(4): 323-332. | Ineligible concept |
| National Institute for Health and Care Excellence (2020) Developing NICE guidelines: The manual. Process and methods [PMG20]. | Ineligible concept |
| National Institute for Health Research (NIHR), Research design service (RDS). Patient and Public Involvement in Health and Social Care Research. National Institute for Health and Care Research; 2018:p 30. | Ineligible concept |
| National Institute for health research, the James Lind alliance Guidebook: version 7, 2018. Available: http://www.jla.nihr.ac.uk/jlaguidebook/downloads/Print-JLA-guidebook-version-7-March-2018.pdf | Ineligible concept |
| National Institute of Health Research. Approaches to public involvement in research. http://www.invo.org.uk/posttyperesource/approaches-to-public-involvement/ | Ineligible concept |
| National Institutes of Health (NIH). Community Engagement. <https://www.nih.gov/health-information/nih-clinical-research-trials-you/community-engagement> | Ineligible concept |
| Nelson E, Lives SO. Impactful engagement and involvement with community stakeholders in the co-production of global health research: How do we make it happen?. | Ineligible concept |
| Ní Shé É, Cassidy J, Davies C, De Brún A, Donnelly S, Dorris E, Dunne N, Egan K, Foley M, Galvin M, Harkin M. Minding the gap: identifying values to enable public and patient involvement at the pre-commencement stage of research projects. Research Involvement and Engagement. 2020 Dec;6:1-0. | Ineligible concept |
| Ní Shé É, Morton S, Lambert V, Ní Cheallaigh C, Lacey V, Dunn E, et al. Clarifying the mechanisms and resources that enable the reciprocal involvement of seldom heard groups in health and social care research: a collaborative rapid realist review process. Heal Expect. (2019) 22:298–306. doi: 10.1111/hex.12865 | Ineligible concept |
| NIHR. Going the extra mile: Improving the nation’s health and wellbeing through public involvement in research 2015. https://www.rds-yh.nihr.ac.uk/wp-content/uploads/2015/06/Going-the-Extra-Mile-Final.pdf. | Ineligible concept |
| Nygaard A, Halvorsrud L, Linnerud S, Grov EK, Bergland A. The James Lind alliance process approach: scoping review. BMJ Open. (2019) 9:1–18. doi: 10.1136/bmjopen-2018-027473 | Ineligible concept |
| Oncode Institute. Patient Engagement Programme. Accessed 04 August, 2021. <https://www.oncode.nl/research/programs/patient-engagement-programme> | Ineligible concept |
| O'Reilly-de Brun M, de Brun T, Okonkwo E, et al. Using Participatory learning & action research to access and engage with ‘hard to reach’ migrants in primary health care research. BMC Health Serv Res 2016; 16: 25. | Ineligible concept |
| Pacer. Youth Advisory Board Toolkit. Accessed December 10, 2023. <https://www.pacer.org/parent/php/php-c245.pdf> | Ineligible concept |
| Pakhale S, Kaur T, Florence K, et al. The Ottawa Citizen Engagement and Action Model (OCEAM): a citizen engagement strategy operationalized through the participatory research in Ottawa, management and point-of-care of tobacco (PROMPT) Study. Res Involv Engagem. 2016; 2(1): 20. | Ineligible concept |
| Pandya-Wood R, Barron DS, Elliott J. A framework for public involvement at the design stage of NHS health and social care research: time to develop ethically conscious standards. Res Involv Engagem. 2017; 3(1): 6. | Duplicate of record from website searching |
| Parry D, Salsberg J, Macauley AC. Guide to researcher and knowledge-user collaboration in health research 2015. Available from: http://www.cihr-irsc.gc.ca/e/44954.html#s2. | Ineligible concept |
| Pérez Jolles MP, Martinez M, Garcia SH, Stein GL, Mentor Parent Group members, Thomas KC. Involving Latina/o parents in patient-centered outcomes research: contributions to research study design, implementation and outcomes. Health Expect. 2017;20:992–1000. | Ineligible concept |
| Phelps D. The voices of young carers in policy and practice. Soc Incl. 2017;5(3):113–21. | Ineligible concept |
| Piil K, Johannessen KB, Pappot H. Strategies for meaningful patient and public involvement in neuro-oncological research. Neuro-Oncology Practice. 2024 Apr 1;11(2):109-10. | Ineligible concept |
| Pinho-Gomes AC, Stone J, Shaw T, Heath A, Cowl J, Norburn L, Thomas V, Scott S. Values, principles, strategies, and frameworks underlying patient and public involvement in health technology assessment and guideline development: A scoping review. International Journal of Technology Assessment in Health Care. 2022 Jan;38(1):e46. | Ineligible concept |
| PMNCH. Global consensus statement on meaningful adolescent and youth engagement. 2020. Available: https://pmnch.who.int/resources/publications/m/item/global-consensus-statement-on-meaningful-adolescent-and-youth-engagement | Ineligible concept |
| Pomey M-P, Lebel P, Clavel N, et al. Development of patient-inclusive teams: toward a structured methodology. Healthc Q. 2018;21:38–44. | Ineligible concept |
| Pomey M-P, Morin E, Neault C et al. Patient advisors: How to implement a process for involvement at all levels of governance in a healthcare organization. Patient Exp J. 2016;3(2):99–112. | Ineligible concept |
| Powell MP, Young AJ, Kim H. A journey in capacity building: revisiting the mullins framework for meaningfully engaging patients in patient centered outcomes research. Front Public Health. 2018; 6: 343. | Ineligible concept |
| Preston J, Lappin E, Ainsworth J, Wood CL, Dimitri P. Involving children and young people as active partners in paediatric health research. Paediatr Child Health. 2024; 34: 11-16. | Ineligible concept |
| Pyne E, Joyce R, Dwyer CP, Hynes SM. Evaluating public and patient involvement in interventional research–a newly developed checklist (EPPIIC) with application to the COB-MS feasibility trial. MedRxiv. 2024:2024-03. | Ineligible concept |
| Rashid A, Thomas V, Shaw T, Leng G. Patient and public involvement in the development of healthcare guidance: an overview of current methods and future challenges. Patient. 2017;10(3):277–82. | Ineligible concept |
| Rawson TM, Castro‐Sánchez E, Charani E, Husson F, Moore LS, Holmes AH, Ahmad R. Involving citizens in priority setting for public health research: implementation in infection research. Health Expectations. 2018 Feb;21(1):222-9. | Ineligible concept |
| Ray KN, Miller E. Strengthening stakeholder-engaged research and research on stakeholder engagement. J Comp Eff Res 2017;6:375–89.doi:10.2217/cer-2016-0096 | Ineligible concept |
| Robinson A. Patient and public involvement: in theory and in practice. The Journal of Laryngology & Otology. 2014 Apr;128(4):318-25. | Ineligible concept |
| Rolfe DE, Ramsden VR, Banner D, Graham ID. Using qualitative health research methods to improve patient and public involvement and engagement in research. Res Involv Engagem. 2018; 4(1): 1-8. | Ineligible concept |
| Rommerskirch-Manietta M, Manietta C, Hoffmann AL, Rohra H, Gove D, Alpers B, Hung L, Geary CR, Abbott KM, Ren LH, Oberfeld S. Participatory development of a framework to actively involve people living with dementia and those from their social network, and healthcare professionals in conducting a systematic review: The DECIDE-SR protocol. Research involvement and engagement. 2023 Jul 11;9(1):52. | Ineligible concept |
| Rowbotham NJ, Smith SJ, Elliott ZC, Leighton PA, Rayner OC, Morley R, et al. Adapting the James Lind alliance priority setting process to better support patient participation: an example from cystic fibrosis. Res Involv Engagem. (2019) 5:1–8. doi: 10.1186/s40900-019-0159-x | Ineligible concept |
| Ruco A, Nichol K. Patient engagement in research and innovation: a new framework. J Med Imaging Radiat Sci 2016;47:290–3.doi:10.1016/j.jmir.2016.10.008 | Ineligible concept |
| Russell J, Greenhalgh T, Taylor M. Patient and public involvement in NIHR research 2006–2019: policy intentions, progress and themes. National Institute for Health Research: Oxford, UK. 2019 Feb. | Ineligible concept |
| Rutgers International. Explore: toolkit instructions and case studies to involve young people as researchers. November 3, 2023. Available: https://rutgers.international/resources/explore-toolkit-instructions/ | Ineligible concept |
| Sauers-Ford HS, Simmons JM, Shah SS, Team HOS. Strategies to engage stakeholders in research to improve acute care delivery. J Hosp Med. 2016;11(2):123–5. | Ineligible concept |
| Scholz B, Bocking J, Happell B. How do consumer leaders co-create value in mental health organisations? Aust Health Rev. 2017;41(5):505–10. | Ineligible concept |
| Scottish Health Council. The Participation Toolkit. 2018 <http://www.scottishhealthcouncil.org/patient__public_participation/participation_toolkit/the_participation_toolkit.aspx#.VZvZybFwbIU> | Ineligible concept |
| Shimmin C, Wittmeier KD, Lavoie JG, Wicklund ED, Sibley KM. Moving towards a more inclusive patient and public involvement in health research paradigm: the incorporation of a trauma-informed intersectional analysis. BMC Health Serv Res. 2017; 17(1): 539. | Ineligible concept |
| Shippee ND, Domecq Garces JP, Prutsky Lopez GJ, et al. Patient and service user engagement in research: a systematic review and synthesized framework. Health Expect 2015;18:1151–66.doi:10.1111/hex.12090 | Ineligible concept |
| Skilton E, Aslam M, Yeung J, Gao F, Melody T. Embedding patient and public involvement within research–How to set up a research patient ambassador group within a NHS trust. Journal of the Intensive Care Society. 2016 Aug;17(3):234-7. | Ineligible concept |
| Smith E, Bélisle-Pipon JC, Resnik D. Patients as research partners; how to value their perceptions, contribution and labor?. Citizen science: theory and practice. 2019 Mar 3;4(1). | Ineligible concept |
| Staats K, Grov EK, Husebø B, Tranvåg O. Framework for patient and informal caregiver participation in research (PAICPAIR): part 1. Adv Nurs Sci. 2020;43(2):E58–70. | Ineligible concept |
| Staats K, Grov EK, Tranvåg O. Framework for Patient and Informal Caregiver Participation in Research (PAICPAIR) Part 2. Advances in Nursing Science. 2024 Apr 1;47(2):188-201. | Ineligible concept |
| Steuli J, Vayena E. The Promising Revolution of Participant-Led Research in Rare Neurological Diseases; Potential Benefits and Pitfalls. Epileptologie; 2016. https://www.researchgate.net/profile/Juerg_Streuli/publication/293769190_The_Promising_Revolution_of_Participant-Led_Research_in_Rare_Neurological_Diseases/links/56bb11cb08ae0a6bc9560d68.pdf. | Ineligible concept |
| Tembo D, Hickey G, Montenegro C, Chandler D, Nelson E, Porter K, Dikomitis L, Chambers M, Chimbari M, Mumba N, Beresford P. Effective engagement and involvement with community stakeholders in the co-production of global health research. bmj. 2021 Feb 16;372. | Ineligible concept |
| The Patient Experience Library. Evidence maps—PPI toolkits analysis. <https://www.patientlibrary.net/cgi-bin/library.cgi?page=Charts;typeId=3> | Ineligible concept |
| Tittlemier BJ, Cooper J, Steliga D, Woodgate RL, Sibley KM. A scoping review to identify and describe the characteristics of theories, models and frameworks of health research partnerships. Health research policy and systems. 2022 Jun 18;20(1):69. | Ineligible concept |
| Todd SA. Patient and Public Involvement (PPI): Developing a conceptual framework from an exploratory study of three healthcare providers (Doctoral dissertation, Loughborough University). | Ineligible concept |
| Townson J, Davies J, Hurt L, Ashfield-Watt P, Paranjothy S. Developing and evaluating a model of public involvement and engagement embedded in a national longitudinal study: HealthWise Wales. International Journal of Population Data Science. 2020;5(3). | Ineligible concept |
| Troya MI, Bartlam B, Chew-Graham CA. Involving the public in health research in Latin America: making the case for mental health. Revista Panamericana de Salud Pública. 2018 May 28;42:e45. | Ineligible concept |
| van Beest W, Boon WP, Andriessen D, Zielhuis M, van der Veen G, Moors EH. How to involve potential users in eHealth innovation: seven strategies from healthcare and design. Design for Health. 2023 Sep 2;7(3):307-25. | Ineligible concept |
| Vanderhout S, Nicholls S, Monfaredi Z, Hampel C, Ashdown L, Bilodeau M, Rich S, Shea B, Fergusson D. Facilitating and supporting the engagement of patients, families and caregivers in research: the “Ottawa model” for patient engagement in research. Research involvement and engagement. 2022 Jun 7;8(1):25. | Ineligible concept |
| Vat LE, Finlay T, Robinson P, Barbareschi G, Boudes M, Diaz Ponce AM, Dinboeck M, Eichmann L, Ferrer E, Fruytier SE, Hey C. Evaluation of patient engagement in medicine development: A multi‐stakeholder framework with metrics. Health Expectations. 2021 Apr;24(2):491-506. | Ineligible concept |
| Walsham M. Involving young londoners: A toolkit for peer research. 2021. <https://3532bf5a-d879-4481-8c8f-127da8c44deb.usrfiles.com/ugd/3532bf_d03284e2369945c09608b9736c381d1e.pdf> | Ineligible concept |
| Warraitch A, Wacker C, Bruce D, Bourke A, Hadfield K. A rapid review of guidelines on the involvement of adolescents in health research. Health Expectations. 2024 Jun;27(3):e14058. | Ineligible concept |
| Weiler‐Wichtl LJ, Leiss U, Gojo J, Kienesberger A, Hansl R, Hopfgartner M, Schneider C. Good to know–This is PPIE! Development of a training tool for public and patient involvement and engagement in pediatric oncological research. Cancer Reports. 2023 Jun;6(6):e1835. | Ineligible concept |
| Williams O, Robert G, Martin GP, Hanna E, O’Hara J. Is co-production just really good PPI? Making sense of patient and public involvement and co-production networks. Decentring health and care networks: reshaping the organization and delivery of healthcare. 2020:213-37. | Ineligible concept |
| Woodward M, Dixon-Woods M, Randall W, Walker C, Hughes C, Blackwell S, Dewick L, Bahl R, Draycott T, Winter C, Ansari A. How to co-design a prototype of a clinical practice tool: a framework with practical guidance and a case study. BMJ Quality & Safety. 2024 Apr 1;33(4):258-70. | Ineligible concept |
| Workman TA. Engaging patients in information sharing and data collection: The role of patient-powered registries and research networks prepared for AHRQ. Available at: <http://www.effectivehealthcare.ahrq.gov/ehc/assets/File/Patient-Powered-Registries-white-paper-130911.pdf> | Ineligible concept |
| World Health Organization. WHO framework for meaningful engagement of people living with noncommunicable diseases, and mental health and neurological conditions. World Health Organization; 2023 May 10. | Ineligible concept |
| World Health Organization. Youth-Centred Digital Health Interventions: A Framework for Planning, Developing and Implementing Solutions With and for Young People. World Health Organization; 2020. <https://www.who.int/publications/i/item/9789240011717> | Ineligible concept |
| Zogas A, Sitter KE, Barker AM, Fix GM, Khanna A, Herbst AN, Vimalananda VG. Strategies for engaging patients in co-design of an intervention. Patient Education and Counseling. 2024 Jun 1;123:108191. | Ineligible concept |

*Exclusions from website searches*

| **Citation** | **Reason for exclusion** |
| --- | --- |
| A guide to working with local communities:  <https://www.publicengagement.ac.uk/resources/report/guide-working-local-communities> | Ineligible concept |
| A quick guide to developing high quality public engagement:  <https://www.publicengagement.ac.uk/resources/guide/quick-guide-developing-high-quality-public-engagement> | Ineligible concept |
| A quick guide to evaluation:  <https://www.publicengagement.ac.uk/resources/guide/quick-guide-evaluation> | Ineligible concept |
| A systems thinking view of public involvement in shaping sexual health policy and services: a short animation:  [A systems thinking view of public involvement in shaping sexual health policy and services: a short animation - Learning for Involvement](https://www.learningforinvolvement.org.uk/content/resource/a-systems-thinking-view-of-public-involvement-in-shaping-sexual-health-policy-and-services-a-short-animation/) | Ineligible concept |
| Accessible and Inclusive Practice Guidelines:  <https://www.publicengagement.ac.uk/resources/practical-tools/accessible-and-inclusive-practice-guidelines> | Ineligible concept |
| An evaluation of a public partnership project between academic institutions and young people with Black African, Asian and Caribbean heritage:  [An evaluation of a public partnership project between academic institutions and young people with Black African, Asian and Caribbean heritage - Learning for Involvement](https://www.learningforinvolvement.org.uk/content/resource/an-evaluation-of-a-public-partnership-project-between-academic-institutions-and-young-people-with-black-african-asian-and-caribbean-heritage/) | Ineligible concept |
| Attributes Framework for Public Engagement:  <https://www.publicengagement.ac.uk/resources/practical-tools/attributes-framework-public-engagement> | Published pre-2013 |
| Attributes Framework for Public Engagement for university staff and students:  <https://www.publicengagement.ac.uk/resources/briefings/attributes-framework-public-engagement-university-staff-and-students> | Duplicate |
| Auditing, benchmarking and evaluating public engagement:  <https://www.publicengagement.ac.uk/resources/guide/auditing-benchmarking-and-evaluating-public-engagement> | Ineligible concept |
| Building research partnerships training (Northern Ireland version):  [Building research partnerships training (Northern Ireland version) - Learning for Involvement](https://www.learningforinvolvement.org.uk/content/resource/building-research-partnerships-training-northern-ireland-version/) | Ineligible concept |
| Community engagement and involvement in global health research – developing a guiding framework:  <https://www.learningforinvolvement.org.uk/content/resource/community-engagement-and-involvement-in-global-health-research-developing-a-guiding-framework/> | Ineligible concept |
| Community-based participatory research: A guide to ethical principles and practice:  <https://www.publicengagement.ac.uk/resources/guide/community-based-participatory-research-guide-ethical-principles-and-practice> | Published pre-2013 |
| COVID-19 and co-production in health and social care research, policy and practice:  [COVID-19 and Co-production in Health and Social Care Research (learningforinvolvement.org.uk)](https://www.learningforinvolvement.org.uk/content/resource/covid-19-and-co-production-in-health-and-social-care-research-policy-and-practice/) | Ineligible study design |
| Creating a supportive culture for public engagement with research:  <https://www.publicengagement.ac.uk/resources/guides/creating-supportive-culture-public-engagement-research> | Ineligible concept |
| Developing a strategy for your Public Engagement support programme:  <https://www.publicengagement.ac.uk/resources/guides/developing-strategy-your-public-engagement-support-programme> | Ineligible concept |
| Enhancing Place-Based Partnerships final report:  <https://www.publicengagement.ac.uk/resources/report/enhancing-place-based-partnerships-final-report> | Ineligible concept |
| High Quality Engagement 101:  <https://www.publicengagement.ac.uk/resources/tutorials/high-quality-engagement-101> | Ineligible concept |
| High Quality Engagement Framework:  <https://www.publicengagement.ac.uk/resources/tools-and-frameworks/high-quality-engagement-framework> | Ineligible concept |
| How to evaluate public engagement projects and programmes:  <https://www.publicengagement.ac.uk/resources/guide/how-evaluate-public-engagement-projects-and-programmes> | Ineligible concept |
| How to evaluate your support for public engagement:  <https://www.publicengagement.ac.uk/resources/guide/how-evaluate-your-support-public-engagement> | Ineligible concept |
| Ignite: Finding and fostering community–university partnerships:  <https://www.publicengagement.ac.uk/resources/report/ignite-finding-and-fostering-community-university-partnerships> | Ineligible concept |
| Learning as an outcome of involvement in research: what are the implications for practice, reporting and evaluation?:  [Learning as an outcome of involvement in research (learningforinvolvement.org.uk)](https://www.learningforinvolvement.org.uk/content/resource/learning-as-an-outcome-of-involvement-in-research-what-are-the-implications-for-practice-reporting-and-evaluation/) | Ineligible concept |
| Mini EDGE tool: Communication:  <https://www.publicengagement.ac.uk/resources/tools-and-frameworks/mini-edge-tool-communication> | Ineligible concept |
| Mini EDGE tool: Leadership:  <https://www.publicengagement.ac.uk/resources/tools-and-frameworks/mini-edge-tool-leadership> | Ineligible concept |
| Mini EDGE tool: Learning:  <https://www.publicengagement.ac.uk/resources/tools-and-frameworks/mini-edge-tool-learning> | Ineligible concept |
| Mini EDGE tool: Mission:  <https://www.publicengagement.ac.uk/resources/tools-and-frameworks/mini-edge-tool-mission> | Ineligible concept |
| Mini EDGE tool: Public:  <https://www.publicengagement.ac.uk/resources/tools-and-frameworks/mini-edge-tool-public> | Ineligible concept |
| Mini EDGE tool: Recognition:  <https://www.publicengagement.ac.uk/resources/tools-and-frameworks/mini-edge-tool-recognition> | Ineligible concept |
| Mini EDGE tool: Support:  <https://www.publicengagement.ac.uk/resources/tools-and-frameworks/mini-edge-tool-support> | Ineligible concept |
| MUPI Partnership Planning:  <https://www.publicengagement.ac.uk/resources/tools-and-frameworks/mupi-partnership-planning> | Ineligible concept |
| NIHR INCLUDE guidance (General):  <https://www.learningforinvolvement.org.uk/content/resource/nihr-include-guidance-general/> | Ineligible concept |
| NIHR public contributor involvement payment policy:  [NIHR public contributor involvement payment policy - Learning for Involvement](https://www.learningforinvolvement.org.uk/content/resource/nihr-public-contributor-involvement-payment-policy/) | Ineligible concept |
| Planning and Evaluation:  <https://www.publicengagement.ac.uk/resources/guide/planning-and-evaluation> | Ineligible concept |
| Reflections on impact and evaluation of public involvement: who, why and what?:  [Reflections on impact and evaluation of public involvement: who, why and what? - Learning for Involvement](https://www.learningforinvolvement.org.uk/content/resource/reflections-on-impact-and-evaluation-of-public-involvement-who-why-and-what/) | Ineligible concept |
| Rough guide: running training for public engagement:  <https://www.publicengagement.ac.uk/resources/report/rough-guide-running-training-public-engagement> | Ineligible concept |
| Social and ethical issues in Public Engagement:  <https://www.publicengagement.ac.uk/resources/guide/social-and-ethical-issues-public-engagement> | Ineligible concept |
| Supporting evaluation:  <https://www.publicengagement.ac.uk/resources/guide/supporting-evaluation> | Ineligible concept |
| Supporting Partnerships:  <https://www.publicengagement.ac.uk/resources/guide/supporting-partnerships> | Ineligible concept |
| The EDGE tool and how to use it:  <https://www.publicengagement.ac.uk/resources/practical-tools/edge-tool-and-how-use-it> | Ineligible concept |
| Tools for talking about public engagement:  <https://www.publicengagement.ac.uk/resources/practical-tools/tools-talking-about-public-engagement> | Ineligible concept |
| UKCPN Principles of Practice:  <https://www.publicengagement.ac.uk/resources/guide/ukcpn-principles-practice> | Ineligible concept |
| Using a logic model to develop your strategy:  <https://www.publicengagement.ac.uk/resources/guide/using-logic-model-develop-your-strategy> | Ineligible concept |
| What influences the involvement of young people in policy making and service improvement? Insights from a map of Scotland’s sexual health system:  [What influences the involvement of young people in policy making and service improvement? Insights from a map of Scotland’s sexual health system - Learning for Involvement](https://www.learningforinvolvement.org.uk/content/resource/what-influences-the-involvement-of-young-people-in-policy-making-and-service-improvement-insights-from-a-map-of-scotlands-sexual-health-system/) | Ineligible concept |
| Working together – guidelines for working effectively on joint projects:  <https://www.learningforinvolvement.org.uk/content/resource/working-together/> | Ineligible concept |
